# SIRT2-mediated deacetylation activates USP22 catalytic function for PD-L1 protein stabilization and tumor immune escape

**DOI:** 10.1172/JCI198270

**Published:** 2026-06-02

**Authors:** Na Li, Qiong Gao, Huijun Jia, Guoqing Xue, Yuanzhang Zhou, Shengnan Wang, Suxian Ma, Bingjin Hu, Zhuoyue Zhao, Chen Su, Yinghong Liu, Wenxuan Xi, Zhonghao Li, Donna D. Zhang, Peng Chu, Zhaolin Sun, Deyu Fang

**Affiliations:** 1 Department of Biochemistry and Molecular Biology, College of Basic Medical Science, Dalian Medical University, Dalian, China.; 2 Department of Pathology, Northwestern University Feinberg School of Medicine, Chicago, Illinois, USA.; 3 Department of Immunology, College of Basic Medical Science,; 4 Dalian College of Pharmacy, and; 5 Institute of Cancer Stem Cell, Dalian Medical University, Dalian, China; 6 Department of Molecular Medicine, Center for Inflammation Science and Systems Medicine, The Herbert Wertheim UF Scripps Institute for Biomedical Innovation & Technology, Jupiter, Florida, USA.

**Keywords:** Immunology, Oncology, Cancer immunotherapy

## Abstract

Immune checkpoint blockade (ICB), including PD-1/PD-L1 inhibitors, has transformed cancer therapy but benefits only a subset of patients. Understanding how PD-L1 is regulated and identifying strategies to overcome resistance remain critical. Here, we identify SIRT2 as a key positive regulator of PD-L1 across multiple human cancers. Unexpectedly, SIRT2 did not act at the transcriptional level but stabilized PD-L1 protein by preventing ubiquitin-mediated degradation. Mechanistically, SIRT2 maintained the protein stability of USP22, a PD-L1 deubiquitinase. Loss of SIRT2 reduced USP22 levels, whereas ectopic USP22 fully rescued PD-L1 expression and reversed the enhanced antitumor immunity induced by SIRT2 inhibition. We further show that SIRT2 directly deacetylates USP22 at K382 and K505 within its catalytic domain, promoting USP22 deubiquitinase activity and protecting both itself and its substrates from degradation. Our findings reveal a molecular mechanism by which an acetylation–deacetylation switch dynamically regulates deubiquitinase catalytic activity. Therapeutically, SIRT2 inhibition synergized with PD-1/PD-L1 blockade and USP22 inhibition to enhance antitumor immunity. Consistently, protein, but not mRNA, levels of SIRT2, USP22, and PD-L1 positively correlated in human bladder cancer and melanoma. Together, these findings define a SIRT2/USP22/PD-L1 axis driving tumor immune evasion and highlight SIRT2 as a promising target to improve ICB efficacy.

## Abstract 2

SIRT2-mediated deacetylation of USP22 prevents its self-ubiquitination to upregulate PD-L1 expression

## Introduction

Bladder cancer is the tenth most prevalent type of human cancers and the 13th most common cause of cancer mortality, with approximately 573,000 new cases and 213,000 deaths worldwide (1, 2). In recent years, the treatment of bladder cancer has made significant progress with the application of immune checkpoint therapy (3–5). Programmed Death Ligand 1 (PD-L1) is a type I transmembrane protein belonging to the Ig B7 family, primarily expressed in tumor cells. PD-L1 binds to the programmed cell death 1 (PD-1) receptor on T lymphocytes, causing the exhaustion of T cells and promoting tumor immune evasion (6, 7). Anti–PD-1/anti–PD-L1 immune checkpoint inhibitors have been approved as clinical treatment of advanced bladder cancer and platinum-resistant metastatic bladder cancer with PD-L1 positivity (8–10). However, PD-1/PD-L1 blockade monotherapy has poor efficacy, with only about 10%–30% of bladder cancer patients responding to PD-1/PD-L1 blockade treatments (10, 11). Therefore, to improve the immunotherapy efficacy of bladder cancer patients, further research on the regulatory mechanisms of PD-L1 and adopting PD-L1/PD-1 inhibitor combined therapy with new targets are important.

SIRT2 is a NAD-dependent type III histone deacetylase and plays an important role in many physiological and pathological processes by modulating various histone and nonhistone substrates (12–14). However, the different expression levels and mechanisms of SIRT2 indicate that its role is complex in different cancers (15). Upregulation of SIRT2 inhibits the transcriptional activity of P53 and promotes the proliferation of lung cancer cells (16). SIRT2 regulates glycolytic metabolism by deacetylating PKM2, leading to the invasion of breast cancer cells (17). Conversely, SIRT2 inhibits APC/C activity by deacetylating CDH1 and CDC20, leading to cell cycle arrest and death in liver cancer cells (18). Hence, SIRT2 has been considered both a tumor promoter and a tumor suppressor. SIRT2 belongs to the sirtuin family, which consists of 7 members (SIRT1–SIRT7) (19). SIRT1, SIRT6, and SIRT7 are mainly located in the nucleus; SIRT3, SIRT4, and SIRT5 are located in the mitochondria; and SIRT2 is mostly located in the cytoplasm, but also shuttles to the nucleus in a cell cycle–dependent manner (20). Recent studies have found that SIRT1 (21), SIRT6 (22), and SIRT7 (23) regulate the expression of PD-L1 through different signaling pathways. However, whether SIRT2 participates in regulating PD-L1 expression and regulatory mechanisms remains unknown.

In this study, we revealed that SIRT2 positively regulates PD-L1 expression through the ubiquitin proteasome pathway in multiple tumors. Using mass spectrometry (MS) and co-IP analyses, we discovered that SIRT2 deacetylated Ubiquitin-Specific Peptidase 22 (USP22) at the K505 residue, which led to increased protein stability by self-deubiquitination. USP22 directly increases PD-L1 stability by deubiquitination (24). Thus, SIRT2-targeted deletion could inhibit tumor immune evasion through the SIRT2/USP22/PD-L1 axis. Notably, the combination of SIRT2 inhibitors and PD-1 antibody synergistically inhibited tumor growth and enhanced antitumor immunity. Our research provides a potential combined therapeutic target for antitumor immune therapy.

## Results

### Identification of SIRT2 as a positive regulator to control PD-L1 protein expression in cancer cells at the posttranscriptional level.

**Figure 1:**
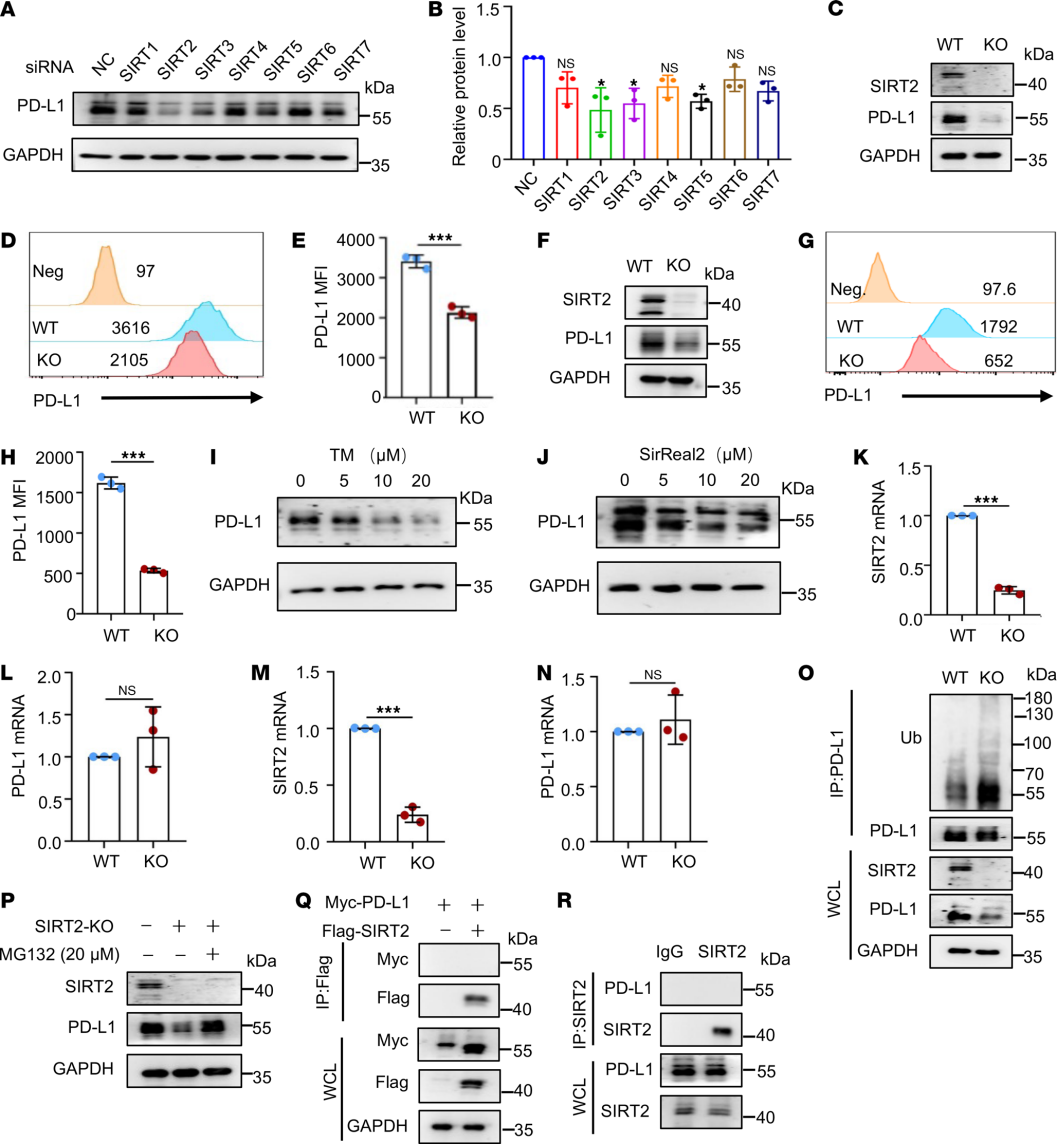
SIRT2 positively regulates PD-L1 expression in multiple cancer cell types. (**A** and **B**) T24 bladder cancer cells were transiently transfected with siRNAs targeting each of the 7 sirtuins. PD-L1 protein expression was assessed by Western blotting. Representative immunoblots are shown in **A**, and quantitative analysis from 3 independent experiments is presented in **B**. (**C**–**E**) CRISPR-mediated KO of SIRT2 in T24 cells was confirmed by Western blot (**C**, top panel). The effect on PD-L1 expression was examined by Western blot (**C**, middle panel) and flow cytometry (**D**, representative plots; **E**, quantified data from 3 independent experiments). (**F**–**H**) The impact of SIRT2 KO on PD-L1 expression was similarly assessed in MB49 murine bladder cancer cells. (**I** and **J**) T24 cells were treated with TM or SirReal2 at each indicated concentration for 8 hours, and PD-L1 expression levels were determined by Western blotting. (**K**–**N**) RT-qPCR analysis of SIRT2 (**K** and **M**) and PD-L1 (**L** and **N**) mRNA levels in T24 and MB49 cells following CRISPR-mediated SIRT2 deletion. WCL, whole-cell lysate. (**O**) Ubiquitination assay of PD-L1 in WT and SIRT2-KO T24 cells. (**P**) Western blot analysis of PD-L1 protein levels in SIRT2 KO T24 cells, with or without treatment with the proteasome inhibitor MG132. (**Q**) Interaction between Myc-tagged PD-L1 and Flag-tagged SIRT2. HEK293T cells were cotransfected with Myc-PD-L1 and Flag-SIRT2, followed by co-IP using an anti-Flag antibody to assess protein interaction. (**R**) Co-IP analysis of endogenous SIRT2 and PD-L1 interaction in T24 cells. Data are shown as mean ± SD. Statistical significance was determined using 1-way ANOVA for **B** and unpaired 2-tailed Student’s *t* test for **E**, **H**, and **K**–**N**. *P < 0.05; ***P < 0.001.

The type III histone deacetylases have been increasingly recognized for their involvement in tumor biology, with members of this family influencing critical oncogenic processes such as cellular growth, proliferation, migration, and invasion (25–27). To investigate these roles, we employed siRNAs to individually silence each of the 7 known sirtuin family members (SIRT1–SIRT7) in T24 human bladder cancer cells. Among these, knockdown of SIRT2 led to a dramatic decrease in the expression of PD-L1, a key immune checkpoint protein known to suppress antitumor immune responses. A modest reduction in PD-L1 expression was also observed upon SIRT3 knockdown (Figure 1, A and B). To further validate the specific role of SIRT2 in PD-L1 regulation, we generated SIRT2-KO human T24 and mouse MB49 bladder cancer cells using a CRISPR/Cas9 approach. Western blotting analysis confirmed the complete SIRT2 deletion (Figure 1, C and F). Consistent with the siRNA data, complete loss of SIRT2 significantly reduced PD-L1 protein levels in both T24 and murine MB49 bladder cancer cells (Figure 1, C and F, middle panels), which was further confirmed by flow cytometry analysis (Figure 1, D, E, G, and H). Consistently, treatment of T24 human bladder cancer cells with the SIRT2-specific inhibitors thiomyristoyl (TM) and SirReal2 resulted in a dose-dependent reduction in PD-L1 expression (Figure 1, I and J).

Western blot analysis revealed a more pronounced decrease in PD-L1 levels upon SIRT2 genetic deletion compared with flow cytometry, suggesting that SIRT2 may regulate intracellular PD-L1 to a greater extent than cell surface expression. To address this possibility, we performed immunofluorescence staining in WT and SIRT2-KO tumor cells, enabling simultaneous assessment of PD-L1 localization in both cytosolic and membrane compartments. These analyses demonstrated that SIRT2 deficiency leads to a more than 70% reduction of PD-L1 in both surface and cytoplasmic compartments in T24 and MB49 tumor cells (Supplemental Figure 1; supplemental material available online with this article; https://doi.org/10.1172/JCI198270DS1). Collectively, these findings indicate that SIRT2 regulates overall PD-L1 protein stability rather than selectively affecting its membrane localization.

Interestingly, real-time RT-PCR analysis did not detect any changes in PD-L1 mRNA expression levels, but confirmed a complete SIRT2 deletion, in both cancer cell lines (Figure 1, K–N). In addition, CRISPR-mediated deletion of SIRT2 led to a substantial reduction in PD-L1 protein, but not its mRNA, expression across multiple cancer cell lines, including human A375 and murine B16 melanoma cells and human endometrial cancer KLE cells (Supplemental Figure 2). These observations strongly suggest that SIRT2 acts as a common positive regulator of PD-L1 expression in cancer cells at a posttranscriptional level.

PD-L1 expression in cancer cells is known to be regulated at both transcriptional and posttranscriptional levels (28, 29). The fact that SIRT2-targeted inhibition reduced PD-L1 protein but not mRNA expression prompted us to explore whether SIRT2 influences PD-L1 expression through modulation of its ubiquitination and proteasomal degradation. Indeed, SIRT2 KO increased PD-L1 ubiquitination in T24 bladder cancer cells (Figure 1O). A similar effect was observed when T24 bladder cancer cells were treated with AGK2, a selective SIRT2 inhibitor, further confirming the role of SIRT2 in suppressing PD-L1 ubiquitination (Supplemental Figure 3). To determine whether this regulation occurs via the proteasome pathway, we treated cells with the proteasome inhibitor MG132, which restored PD-L1 protein levels in SIRT2-deficient cells to levels comparable with controls (Figure 1P). These findings collectively suggest that SIRT2 maintains PD-L1 protein stability by protecting it from ubiquitin-mediated proteasomal degradation.

We then determined whether SIRT2 exerts its effect through direct interaction with PD-L1 using co-IP experiments. However, Western blot analysis failed to detect any physical interaction between SIRT2 and PD-L1 either in transiently transfected HEK293T cells or endogenously in T24 cells (Figure 1, Q and R). These results suggest that SIRT2 likely regulates PD-L1 indirectly, potentially through modulation of upstream components of the protein degradation machinery.

### SIRT2 is a de novo USP22 deacetylase.

**Figure 2:**
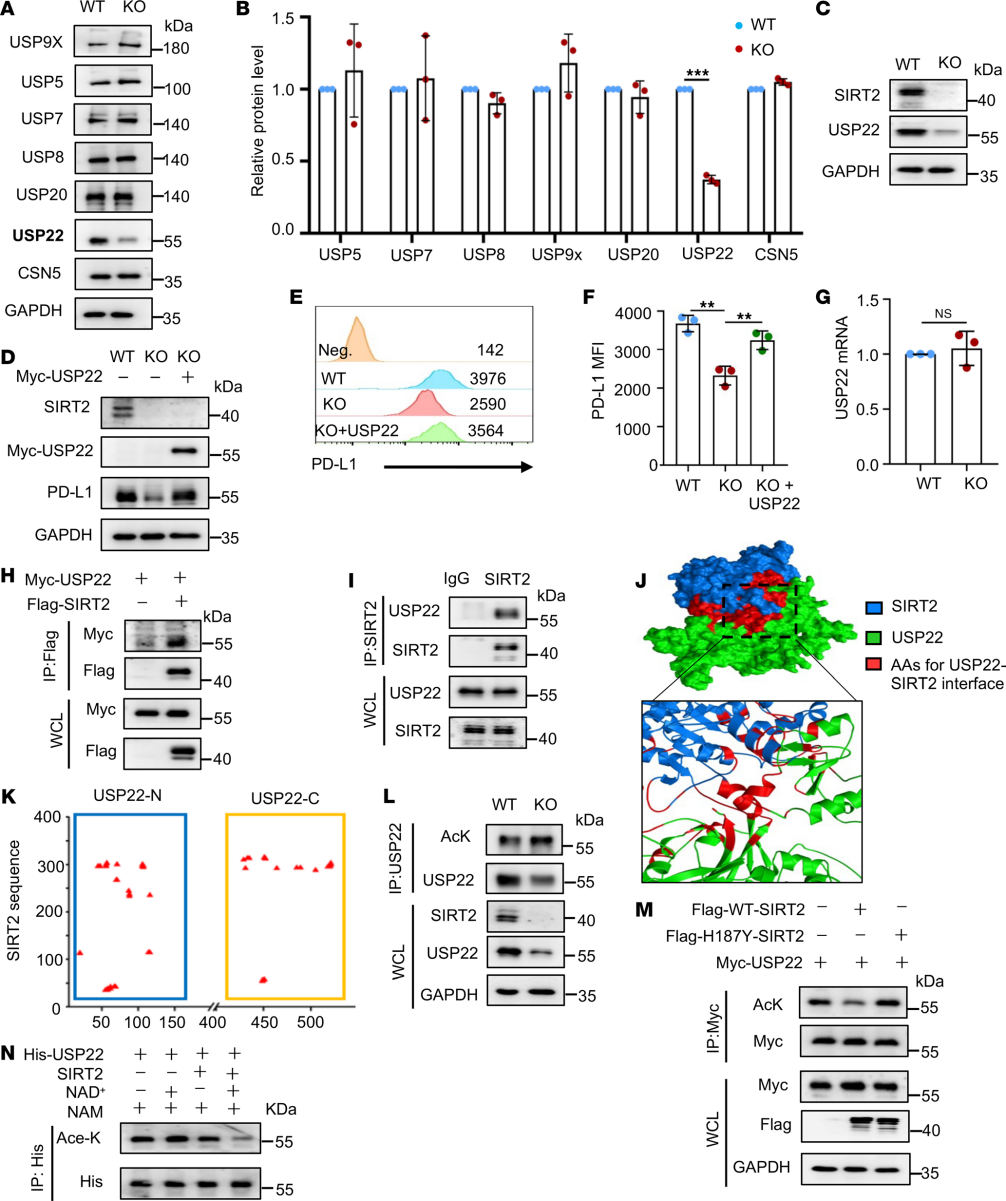
SIRT2 inhibits PD-L1 ubiquitination by regulating USP22. (**A**) Western blot analysis of deubiquitinases in SIRT2-KO T24 cells, including USP9X, USP5, USP7, USP8, USP20, USP22, and CSN5. (**B**) Quantification of PD-L1 expression. (**C**) Western blot analysis of USP22 protein levels in SIRT2-KO MB49 cells. (**D**–**F**) SIRT2-KO T24 cells were transfected with or without Myc-USP22, and PD-L1 expression was assessed by Western blotting and flow cytometry 36 hours after transfection. (**G**) RT-qPCR analysis of USP22 mRNA levels in WT and SIRT2-KO T24 cells. (**H**) Analysis of the interaction between Myc-USP22 and Flag-SIRT2 in HEK293T cells. (**I**) Co-IP analysis of endogenous interaction between SIRT2 and USP22 in T24 cells. (**J** and **K**) Structural modeling of SIRT2-USP22 interaction. (**J**) Simulated interaction diagram. (**K**) Dot plot representing predicted interacting amino acid residues. Blue box: N-terminal region of USP22; orange box, C-terminal region of USP22. (**L**) Analysis of endogenous USP22 acetylation in WT and SIRT2-KO T24 cells. (**M**) USP22 acetylation was detected by immunoprecipitating with anti-Myc antibodies followed by blotting with anti–pan-acetylation antibodies. (**N**) In vitro analysis of SIRT2 deacetylation of acetyl-USP22. Data are shown as mean ± SD. Statistical significance was determined using unpaired 2-tailed Student’s *t* test for **B** and **G** and 1-way ANOVA for **F**. **P < 0.01; ***P < 0.001.

Several deubiquitinating enzymes, including USP2 (30), USP5 (31), USP7 (32), USP8 (33), USP9X (34), USP10 (35), USP19 (36), USP20 (37), USP22 (38), and CSN5 (39), deubiquitinated and stabilized PD-L1 protein in cancer cells. Interestingly, SIRT2 KO downregulated the expression of USP22, but not any others, in T24 cells (Figure 2, A and B, and Supplemental Figure 4), suggesting that SIRT2 specifically stabilizes the PD-L1 deubiquitinase USP22. Interestingly, we observed a modest but statistically significant increase in USP19 expression, suggesting a potential compensatory response to reduced USP22 levels in SIRT2-KO tumor cells. Similar trends were observed across multiple cell lines, including MB49, A375, and B16 (Figure 2C and Supplemental Figure 4). Consistently, pharmacological SIRT2 inhibition using specific inhibitors, AGK2, TM, and SirReal2, resulted in a dose-dependent reduction in USP22 protein expression (Supplemental Figure 5). In contrast, CRISPR-mediated deletion of Sirt1 or Sirt3, both known regulators of PD-L1 expression (40–42), had no detectable effect on USP22 levels in human bladder cancer cells (Supplemental Figure 6). These results indicate that SIRT2 promotes PD-L1 through upregulating the PD-L1–specific deubiquitinase USP22 expression. Supporting this speculation, USP22 overexpression fully rescued PD-L1 protein levels in SIRT2-KO cells (Figure 2, D–F). Interestingly, RT-qPCR did not detect any meaningful changes in USP22 mRNA by SIRT2 KO in T24 cells, A375 cells, and SIRT2-knockdown KLE cells (Figure 2G and Supplemental Figure 7). Collectively, these results indicate that SIRT2 upregulates USP22 expression at the posttranscriptional level.

To elucidate the molecular mechanism by which SIRT2 regulates USP22, we first examined whether these proteins physically interact. Co-IP assays revealed that SIRT2 directly interacts with USP22 in transiently transfected HEK293T cells (Figure 2H). The interaction of endogenous SIRT2 with USP22 was also confirmed at the endogenous level in T24 human bladder cancer cells (Figure 2I). Further analysis with molecular dynamics (MD) simulations and molecular mechanics/Poisson-Boltzmann surface area (MM/PBSA) binding free energy calculations demonstrated that SIRT2 binds USP22 with high affinity, with a calculated binding energy of –4,642.321 kJ/mol (Supplemental Table 1). MD simulations also identified 40 pairs of highly probable interacting amino acids between SIRT2 and USP22 (Figure 2K). Structural modeling further confirmed that these interacting residues are in close spatial proximity, suggesting a stable and specific interaction interface (Figure 2J). These results confirm the physical interaction between SIRT2 and USP22 proteins in cancer cells.

As a class III histone deacetylase, SIRT2 is known to target nonhistone proteins for deacetylation (43). Consistent with this, both CRISPR-mediated SIRT2 KO and pharmacological inhibition with AGK2 led to a more than 2-fold increase in USP22 acetylation in T24 cancer cells (Figure 2L and Supplemental Figure 8A). Conversely, overexpression of SIRT2, but not its catalytically inactive mutant, reduced USP22 acetylation (Figure 2M). These results indicate that SIRT2 directly deacetylates USP22 and that its enzymatic activity is essential for this function. To support this conclusion, in vitro deacetylation assays showed that USP22 acetylation was reduced only in the presence of both NAD^+^ and SIRT2, but not with NAD^+^ or SIRT2 alone, confirming that SIRT2 directly and specifically deacetylates USP22 in a NAD^+^-dependent manner (Figure 2N).

### SIRT2 protects USP22 from ubiquitination-induced degradation.

**Figure 3:**
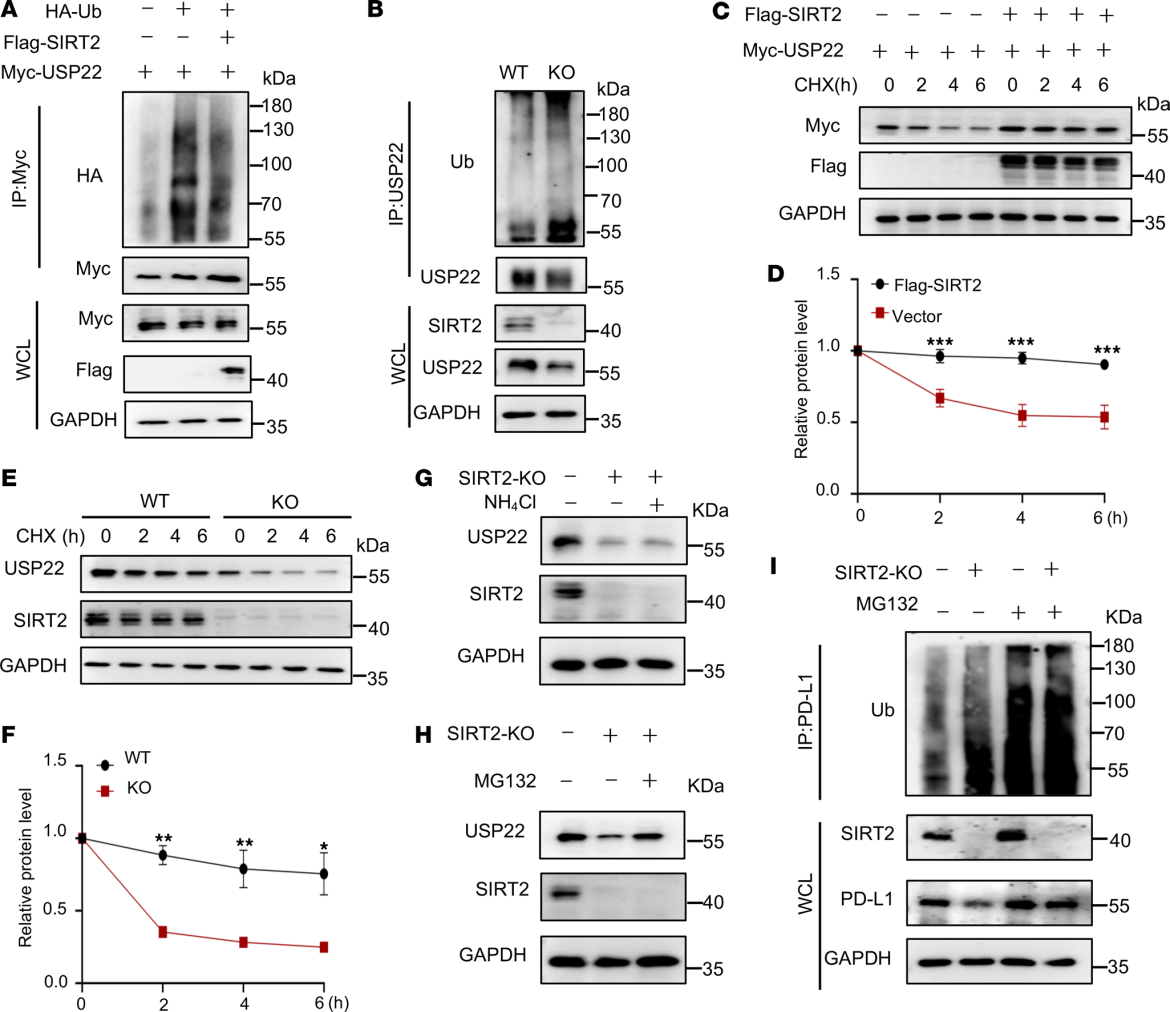
SIRT2 promotes USP22 ubiquitination. (**A**) USP22 ubiquitination was detected by IP using anti-Myc antibodies and immunoblotting with anti-HA antibodies. (**B**) Ubiquitination assay of USP22 in WT and SIRT2-KO T24 cells. (**C** and **D**) Stability assay of Myc-USP22 in HEK293T cells cotransfected with or without Flag-SIRT2 treated with CHX. (**E** and **F**) Stability assay of endogenous USP22 in WT and SIRT2-KO T24 cells following CHX treatment. (**G**–**I**) WT and SIRT2-KO cells were treated with NH_4_Cl (**G**) or MG132 (**H** and **I**). USP22 and SIRT2 expression and PD-L1 ubiquitination were determined. Data are shown as mean ± SD. Statistical significance was determined using unpaired 2-tailed Student’s *t* test for **D** and 1-way ANOVA for **F**. *P < 0.05; **P < 0.01; ***P < 0.001.

While competition between acetylation and ubiquitination on the same lysine (K) residues is a known mechanism of protein stability regulation (44–46), this does not appear to be the case here because SIRT2 inhibition increased both the acetylation (Figure 2M) and ubiquitination (Figure 3A) of USP22, suggesting that these modifications may not act through competition with each other. Supporting this, SIRT2 KO or pharmacological inhibition by AKG2 increased USP22 ubiquitination levels (Figure 3B and Supplemental Figure 8B).

Given that SIRT2 loss enhances USP22 ubiquitination, we next assessed whether SIRT2 affects USP22 protein stability. Indeed, SIRT2 expression significantly prolonged the half-life of USP22 in HEK293T cells (Figure 3, C and D). Conversely, both CRISPR-mediated SIRT2 deletion and AGK2 treatment accelerated USP22 degradation in cancer cells (Figure 3, E and F, and Supplemental Figure 8, C and D). The lysosomal inhibitor NH_4_Cl failed to rescue USP22 expression in SIRT2-KO tumor cells (Figure 3G). In contrast, treatment of SIRT2-KO T24 cells with the proteasomal inhibitor MG132 largely restored both USP22 and PD-L1 protein levels (Figure 3, H and I). We also observed that the proteasomal inhibitor MG132 induced a pronounced accumulation of ubiquitinated PD-L1, as well as total PD-L1 protein levels in both WT and SIRT2-KO cells (Figure 3I). Taken together, these findings demonstrate that SIRT2 stabilizes USP22 by deacetylating it, thereby protecting USP22 from ubiquitin-mediated proteasomal degradation. Through this mechanism, SIRT2 indirectly promotes PD-L1 protein expression, positioning SIRT2 as a critical upstream regulator of tumor immune evasion.

### SIRT2 specifically deacetylates USP22 at lysine residues in its deubiquitinase catalytic domain to control deubiquitination activity.

**Figure 4:**
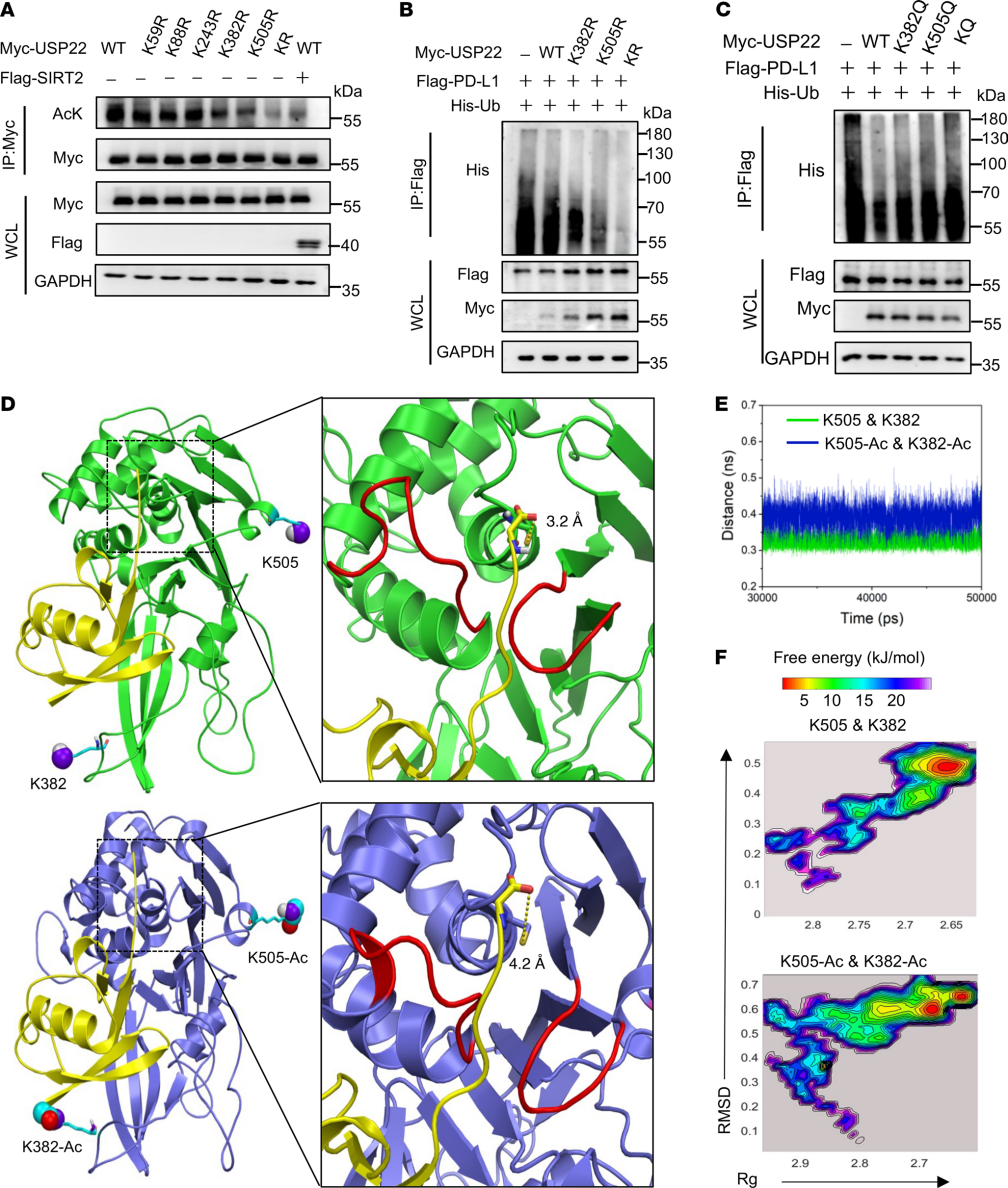
SIRT2-mediated deacetylation alters USP22 interaction. (**A**) Myc-tagged WT USP22 and its mutant by replacing each identified lysine with arginine (KR) were cotransfected into HEK293T cells with or without Flag-tagged SIRT2. USP22 acetylation was determined. (**B**) His-tagged ubiquitin and Flag–PD-L1 were cotransfected into HEK293T cells with or without Myc-USP22 and its K382R, K505R, or double mutant (dKR). PD-L1 ubiquitination was detected. (**C**) The effect of USP22 K382Q, K505Q, and dKQ mutants on PD-L1 ubiquitination was analyzed as in **B**. (**D**) Schematic diagram showing the ubiquitin-binding and acetylation sites of USP22 at K505 and K382. (Enlarged images are in Supplemental Figure 5A.) (**E**) Superimposition of the initial UB-USP22 complex structure with conformations obtained after 50 ns MD simulations for K505-, K382-, and double-acetylated (K505 and K382) UB-USP22 complexes. (**F**) Energy landscape plots of the UB-USP22 complexes with different acetylation states, generated from 50 ns MD simulations. RMSD, root mean square deviation.

To further elucidate the molecular mechanism by which SIRT2-mediated deacetylation stabilizes USP22 protein in cancer cells, we performed IP-MS analysis to identify acetylated lysine residues on USP22 that may serve as SIRT2 substrates. This analysis revealed 5 acetylated lysine residues, including K59, K88, K243, K382, and K505, that could potentially be deacetylated by SIRT2 (Supplemental Figure 9). To determine which residues are functionally relevant targets of SIRT2, we generated a panel of USP22 mutants, each with one of these lysines substituted by arginine (R). Among the 5 mutants, K505R and K382R showed a pronounced reduction in USP22 acetylation, but mutation of any of the remaining 3 lysine residues did not reduce USP22 acetylation. When both K382 and K505 were mutated, USP22 acetylation levels were largely diminished (Figure 4A), identifying 2 lysine residues as dominant acetylation sites.

Notably, both K382 and K505 reside within the catalytic domain of USP22, suggesting that acetylation at these residues may influence the enzyme’s deubiquitinating activity. We then performed functional assays to test this hypothesis. Expression of either the K382R or K505R mutant significantly enhanced USP22 deubiquitinase activity, as evidenced by their stronger suppression of PD-L1 ubiquitination (Figure 4B). In contrast, acetylation-mimicking mutants, where K382 and K505 were replaced by glutamine (Q), lost this activity, resulting in diminished ability to inhibit PD-L1 ubiquitination (Figure 4C). These results clearly indicate that acetylation of the 2 lysine residues in the enzymatic pocket of USP22 inhibits its catalytic activity.

Analysis of 50 ns MD simulations revealed that lysine acetylation at positions K505, K382, or both induces notable conformational changes in the catalytic domain of USP22, particularly within loops 253–262 and 472–478, compared with the unacetylated form. Given the critical role of C185 in catalysis, the distance between its sulfur atom and the oxygen atom of the C-terminal glycine of ubiquitin was monitored over the final 20 ns of each trajectory. The average distances were 3.2 Å (unacetylated), 4.0 Å (505K-Ac), 4.0 Å (382K-Ac), and 4.2 Å (double acetylated), indicating that the acetylation relocates the C-terminal glycine of ubiquitin away from the catalytic core of USP22. These findings suggest that lysine acetylation induces structural USP22 rearrangements that cause loop 253–262 and 472–478 movements to sterically hinder ubiquitin access to the catalytic pocket, thereby impairing USP22’s deubiquitinase activity (Figure 4, D and E, and Supplemental Figure 10, A and B). Further MM/PBSA binding free energy calculations were performed to compare nonacetylated and acetylated forms of USP22 in complex with ubiquitin. After a 50 ns simulation, nonacetylated UB-USP22 adopted a stable conformation within a single energy well, indicating strong and stable interaction. In contrast, acetylated forms, including UB-USP22(K382Ac), UB-USP22(K505Ac), and UB-USP22(K382Ac & K505Ac), displayed multiple energy wells, suggesting conformational instability and impaired ubiquitin binding (Figure 4, E and F, and Supplemental Figure 10C). Supporting this, MM/PBSA analysis showed that acetylated USP22 variants had significantly weaker binding energies with ubiquitin, with dual acetylation at K382 and K505 producing the most severe loss of binding affinity (Supplemental Table 2). These findings indicate that SIRT2 enhances USP22 catalytic activity by deacetylating K382 and K505, thereby stabilizing its conformation and ubiquitin-binding efficiency.

**Figure 5:**
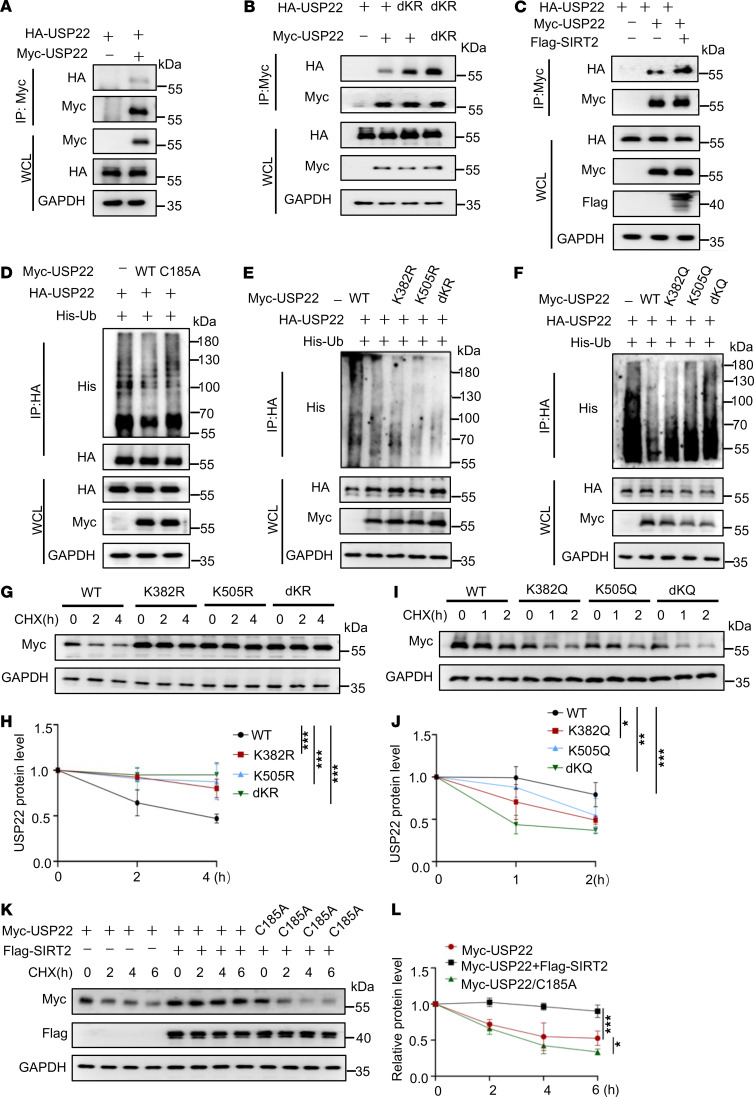
SIRT2-mediated deacetylation inhibits USP22 self-deubiquitination. (**A**) Analysis of USP22 homodimerization in HEK293 cells cotransfected with HA- and Myc-tagged USP22. (**B**) Analysis of USP22/KR mutant interactions as in **A**. (**C**) Interaction between HA-USP22 and Myc-USP22 with or without Flag-SIRT2 in HEK293T cells. (**D**) His-ubiquitin and HA-USP22 were cotransfected into HEK293T cells with Myc-USP22 or Myc-C185A-USP22. USP22 ubiquitination was immunoprecipitated with anti-HA antibodies and detected with anti-His antibody. (**E** and **F**) The effect of KR (**E**) or KQ (**F**) mutations on USP22 ubiquitination was analyzed as in **D**. (**G**–**L**) The effect of KR (**G** and **H**), KQ (**I** and **J**), or CA (**K** and **L**) mutations on USP22 protein stability was analyzed. Stability in transiently transfected HEK293T cells treated with CHX for the indicated time points. Western blot analysis was used to assess protein levels, and USP22 levels normalized to GAPDH were quantified. Data are shown as mean ± SD, *N* = 3. Statistical significance was determined using unpaired 2-tailed Student’s *t* test. *P < 0.05; **P < 0.01; ***P < 0.001.

To further confirm whether SIRT2-mediated deacetylation enhances USP22 activity, we analyzed the effect of USP22 acetylation on its auto-deubiquitination, a mechanism critical for USP22 self-stabilization. A prior study suggested that USP22 can form homodimers (BioGRID) (47). To test this, we performed co-IP in HEK293T cells cotransfected with HA- and Myc-tagged USP22 and confirmed USP22-USP22 interaction (Figure 5A). Interestingly, mutation of the critical lysine residues K382 and K505 increased USP22 homodimerization (Figure 5B), suggesting that acetylation at these sites restrains dimer formation and that SIRT2-mediated deacetylation promotes this process. Consistently, SIRT2 overexpression enhanced USP22 self-interaction (Figure 5C), further supporting a model in which SIRT2 facilitates USP22 dimerization and functional activity.

Next, we determined how SIRT2 stabilizes USP22 protein in cancer cells. WT USP22, but not the catalytically inactive C185A mutant, reduced its ubiquitination, confirming that auto-deubiquitination requires catalytic activity (Figure 5D). Importantly, compared with that of WT USP22, coexpression of the K382R and K505R mutants further enhanced suppression of its auto-ubiquitination (Figure 5E), whereas the K382Q or K505Q mutant totally abolished this self-deubiquitination effect (Figure 5F). These results suggest that acetylation at K382 and K505 negatively regulates USP22 auto-deubiquitination. Consequently, coexpression of the K382R and K505R mutants significantly prolonged USP22 half-life, whereas K382Q and K505Q mutants failed to do so (Figure 5, G–J), indicating that acetylation at these sites reduces USP22 stability by promoting its degradation via ubiquitination. Indeed, SIRT2 overexpression did not stabilize the catalytically inactive C185A mutant, suggesting that SIRT2-mediated stabilization of USP22 depends on USP22’s own enzymatic activity (Figure 5, K and L).

Collectively, these findings reveal a novel mechanism by which SIRT2 stabilizes USP22 through direct deacetylation of K382 and K505, promoting auto-deubiquitination and protein stability and ultimately enhancing PD-L1 deubiquitination. This SIRT2/USP22 axis provides an important regulatory pathway for immune checkpoint control in cancer cells.

### Targeted SIRT2 inhibition enhances antitumor immunity.

**Figure 6:**
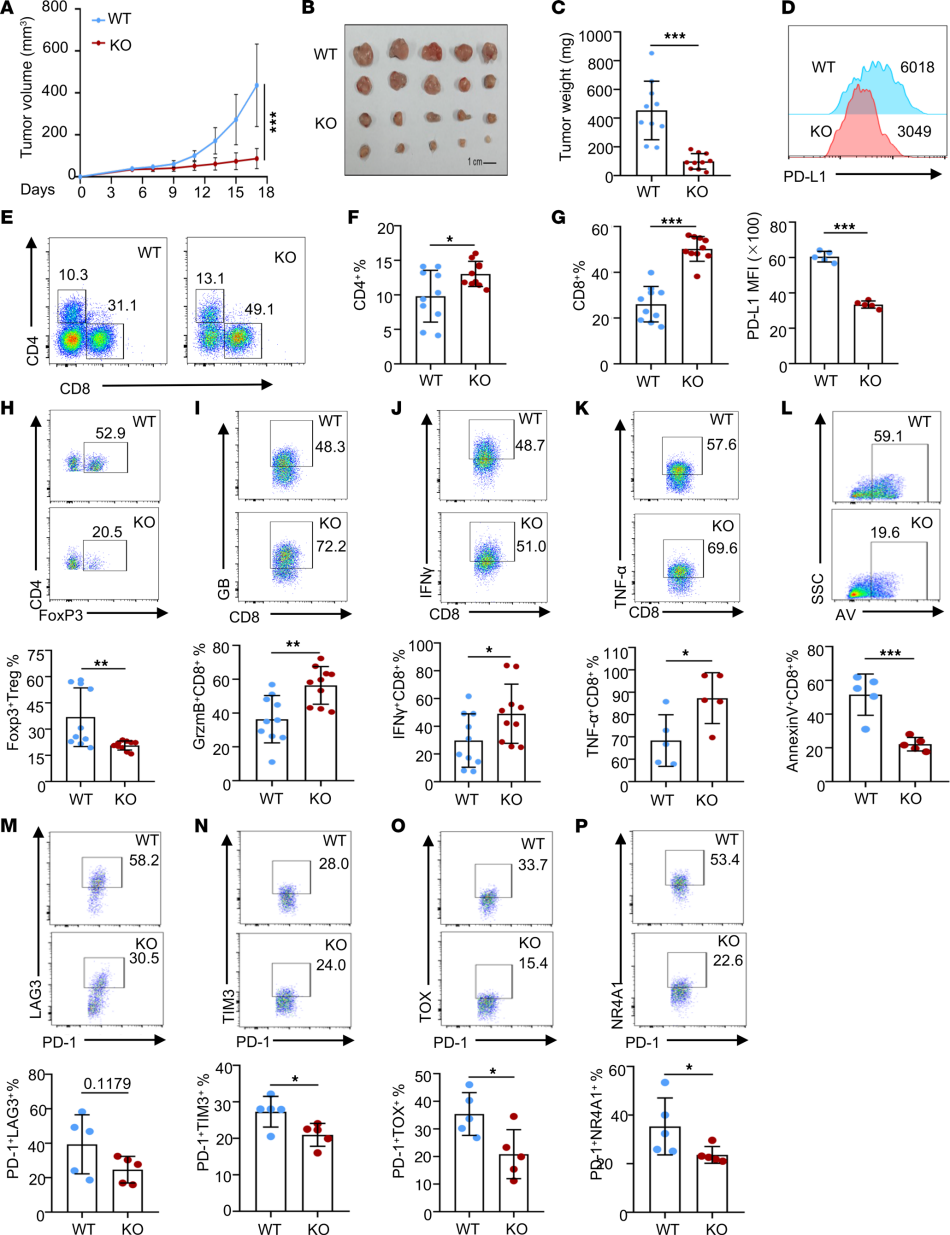
SIRT2 KO enhances antitumor immunity. (**A**–**C**) WT and SIRT2-KO MB49 cells were subcutaneously implanted into the right flanks of C57BL/6J mice (*n* = 10). Representative images of mouse tumors (**B**), tumor volume (**A**), and tumor weight (**C**). Scale bar: 1 cm. (**D**) PD-L1 expression of CD45^–^ tumor cells (*n* = 10). (**E**) Analysis of CD4^+^ and CD8^+^ T cells in a gated CD45^+^ population. (**F** and **G**) Quantification of CD4^+^ (**F**) and CD8^+^ (**G**) T cells, shown as percentages of tumor-infiltrating lymphocytes in MB49 tumors. (**H**) Analysis of CD4^+^FoxP3^+^ Treg cells as a percentage of tumor-infiltrating CD4^+^ T cells. (**I**–**L**) Analysis of granzyme B^+^ (**I**), IFN-γ^+^ (**J**), and TNF-α^+^ (**K**) CD8^+^ T cells and expression of annexin V (**L**) CD8^+^ T cells as percentages of tumor-infiltrating CD8^+^ T cells in MB49 tumors (*n* = 10). (**M**–**P**) The expression levels of TIM-3, LAG-3, Tox, and NR4A1 on the gated intratumoral CD8^+^PD-1^+^ T cells were analyzed. Representative flow images (top panels) and data from 5 pairs of mice are shown. Data are shown as mean ± SD. Statistical significance was determined using 1-way ANOVA for **A** and unpaired 2-tailed Student’s *t* test for the remaining panels. *P < 0.05; **P < 0.01; ***P < 0.001.

PD-L1 expressed in tumor cells plays a key role in immune evasion by binding to PD-1 receptors on immune cells, thereby suppressing antitumor immune responses (48, 49). Our discovery that inhibition of SIRT2 reduced PD-L1 protein expression on the surface of tumor cells provided a rationale for targeting SIRT2 potentiating antitumor immunity. Consistent with this hypothesis, selective inhibition of SIRT2 in tumor cells significantly impaired syngeneic tumor growth (Figure 6, A–C). Mechanistically, SIRT2 deletion led to a pronounced decrease in PD-L1 expression in CD45^+^ tumor cells (Figure 6D). Importantly, tumors derived from SIRT2-KO cells showed increased infiltration of CD4^+^ and CD8^+^ T cells (Figure 6, E–G), along with a dramatic reduction in immunosuppressive FoxP3^+^ Tregs (Figure 6H). Notably, SIRT2 loss dramatically enhanced CD8 T cell cytotoxic function, as indicated by elevated levels of granzyme B, IFN-γ, and TNF-α (Figure 6, I–K). In contrast, the proportion of terminally exhausted/apoptotic (annexin V^+^) CD8^+^ T cells was significantly decreased in SIRT2-deficient tumors (Figure 6L).

Elevated PD-L1 expression promotes and sustains CD8^+^ T cell exhaustion, particularly under conditions of chronic antigen exposure, such as cancer or persistent viral infection (50). Consistent with this paradigm, the frequencies of PD-1^+^LAG3^+^ and PD-1^+^TIM-3^+^CD8^+^ T cells were significantly reduced in SIRT2-deficient tumors (Figure 6, M and N). Further analysis of TOX and NR4A1, both of which are critical for CD8^+^ T cell exhaustion, confirmed that SIRT2 inhibition reduced the PD-1^+^TOX^+^ and PD-1^+^NR4A1^+^ exhausted intratumoral CD8^+^ T cells (Figure 6, O and P). These findings suggest that the deletion of SIRT2 in tumor cells enhances antitumor immune responses primarily by relieving PD-L1–mediated suppression of CD8^+^ T cell function.

This immunostimulatory effect of SIRT2 inhibition was further validated in the B16 melanoma model (Supplemental Figure 11, A–C). Compared with WT B16 cells, SIRT2-KO B16 tumors exhibited significantly reduced PD-L1 expression in CD45^–^ tumor cells (Supplemental Figure 11D). This was accompanied by increased infiltration of CD4^+^ and CD8^+^ T cells (Supplemental Figure 11, E–G) and a reduction in FoxP3^+^ Tregs (Supplemental Figure 11H). Enhanced cytotoxicity of CD8^+^ T cells was also observed, as evidenced by increased IFN-γ and granzyme B levels (Supplemental Figure 11, I and J) and a reduction in the cell surface PD-1 expression and exhausted annexin V^+^ phenotype (Supplemental Figure 11, K and L). Notably, SIRT2 deletion had no effect on the intrinsic proliferative capacity of MB49 cells in vitro (Supplemental Figure 12A). Furthermore, tumor growth of WT and SIRT2-KO MB49 cells was comparable in immunocompromised mice, confirming that the antitumor effects of SIRT2 loss are largely immune dependent (Supplemental Figure 12, B–D). Together, these results support that SIRT2 inhibition amplifies PD-L1–regulated antitumor immune responses across different tumor types.

### SIRT2 evades CD8^+^ T cell antitumor immunity through USP22-mediated upregulation of PD-L1.

**Figure 7:**
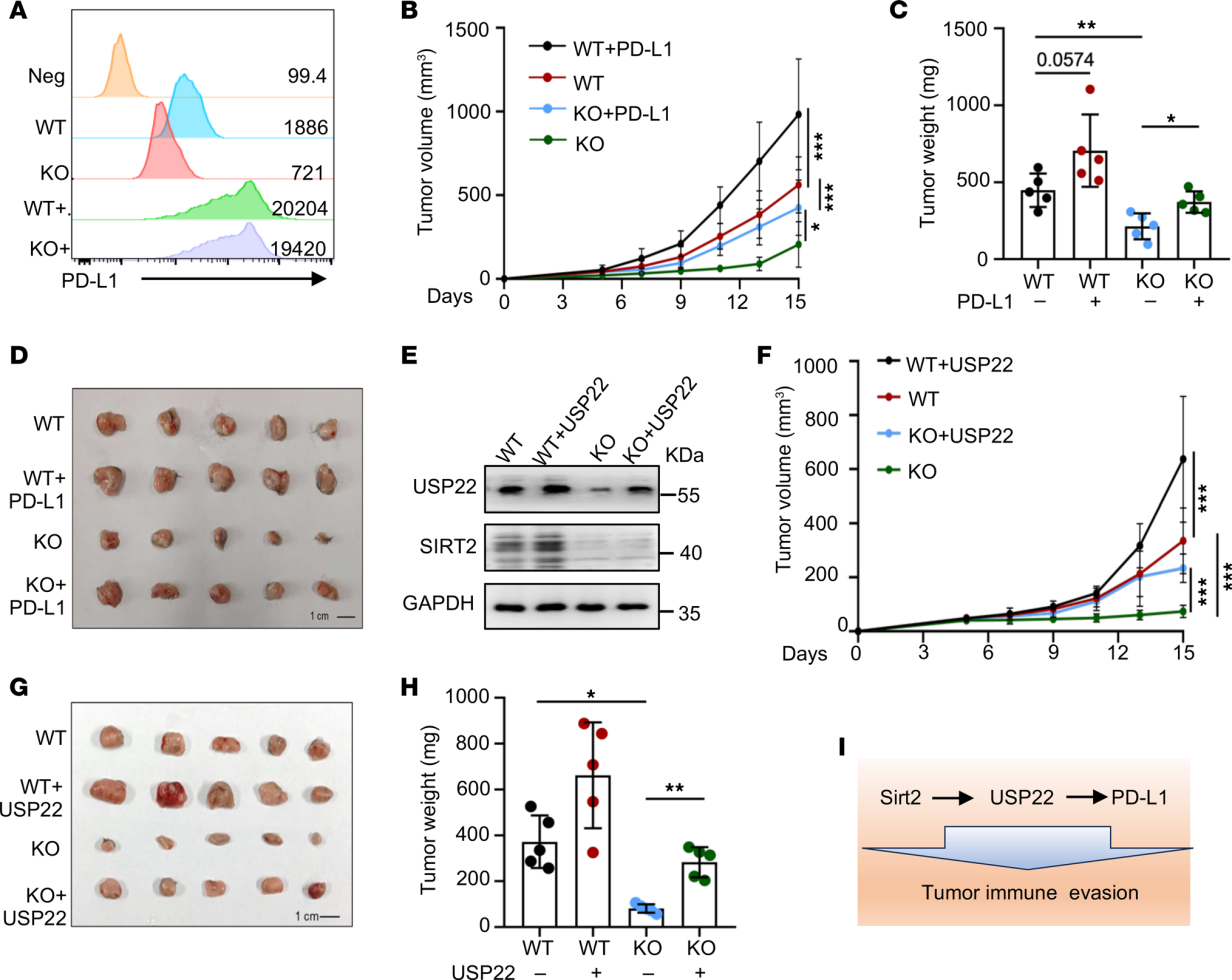
SIRT2 KO enhances antitumor immunity through downregulating PD-L1. (**A**–**D**) WT, SIRT2-KO, and PD-L1–reconstituted WT or SIRT2-KO MB49 cells were implanted subcutaneously into the right flanks of C57BL/6J mice (*n* = 5). The PD-L1 expression levels (**A**), tumor volume (**B**), tumor weight (**C**), and representative tumor images (**D**) are shown. (**E**–**H**) WT, SIRT2-KO, and USP22-reconstituted WT or SIRT2-KO MB49 cells were implanted subcutaneously into the right flanks of C57BL/6J mice (*n* = 5). The USP22 expression levels (**E**), tumor volume (**F**), representative tumor images (**G**), and tumor weight (**H**) are shown. (**I**) A proposed pathway for SIRT2 in tumor immune evasion. Scale bars: 1 cm. Data are shown as mean ± SD. Statistical significance was determined using 1-way ANOVA. *P < 0.05; **P < 0.01; ***P < 0.001.

Our studies uncover a novel mechanism by which SIRT2 promotes immune evasion from CD8^+^ T cell–mediated antitumor immunity, at least in part, through USP22-dependent upregulation of PD-L1. Reexpression of PD-L1 in SIRT2-deficient tumor cells largely restored tumor growth (Figure 7, A–D). We also observed slightly lower PD-L1 levels in SIRT2-KO tumor cells compared with WT cells under ectopic expression conditions, likely due to loss of SIRT2-mediated protection of PD-L1 from ubiquitination-dependent degradation (Figure 7A). Collectively, our data indicate that the protumorigenic function of SIRT2 is primarily mediated through its regulation of PD-L1 expression.

Next, we stably overexpressed USP22 in both WT and SIRT2-deficient MB49 tumor cells to determine whether, similar to PD-L1, USP22 reconstitution could reverse the antitumor effects of SIRT2 inhibition (Figure 7E). As expected, enforced USP22 expression significantly accelerated tumor growth in WT MB49 cells, further supporting its oncogenic role. Importantly, stable reconstitution of USP22 in SIRT2-null MB49 cells largely restored tumor growth to levels comparable to WT tumors (Figure 7, F–H). Similar to PD-L1, USP22 levels were modestly reduced in SIRT2-KO tumor cells compared with WT cells under ectopic expression conditions, likely due to loss of SIRT2-mediated protection of USP22 from ubiquitination-dependent degradation (Figure 7E). These findings indicate that SIRT2 inhibition enhances tumor rejection primarily through downregulation of USP22 protein expression, thereby attenuating USP22-mediated PD-L1 upregulation and tumor immune evasion (Figure 7I). Therefore, targeted deletion or inhibition of SIRT2 enhances antitumor immunity by downregulating PD-L1, boosting T cell infiltration and function, and reversing T cell exhaustion, highlighting SIRT2 as a promising immunotherapeutic target in cancer.

### SIRT2 inhibition synergizes with anti–PD-1 immune checkpoint blockade to potently suppress tumor growth.

**Figure 8:**
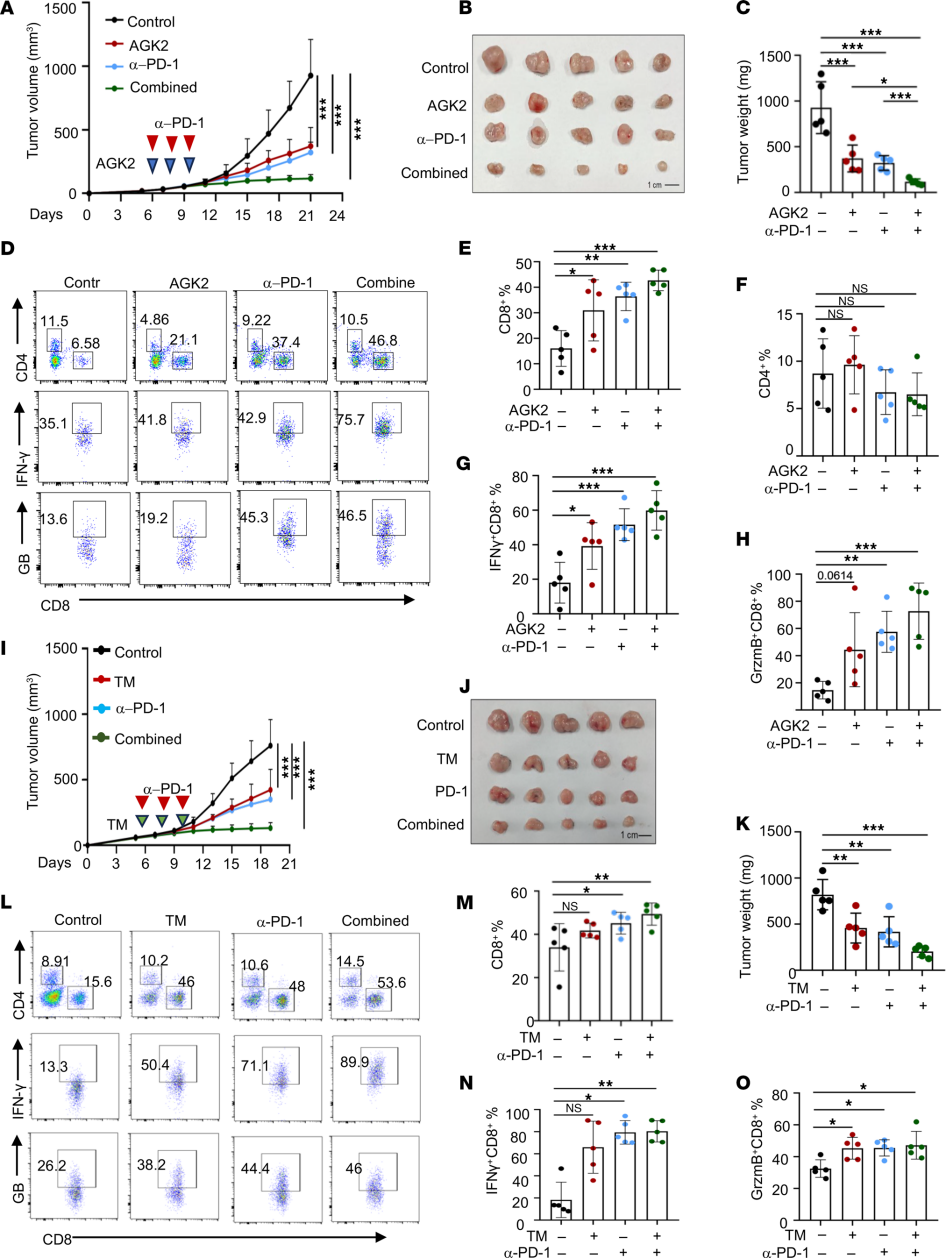
SIRT2 blockade combined with anti–PD-1 therapy effectively inhibited tumor growth. (**A**–**C**) MB49 cells were implanted subcutaneously into the right flanks of C57BL/6J mice (*n* = 5). Mice were treated with AGK2 (10 mg/kg), PD-1 antibody (50 μg per mouse), IgG2a isotype control (50 μg per mouse), or a combination via intraperitoneal injection on day 7. Tumor growth (**A**), tumor images (**B**), and tumor weight (**C**) are shown. (**D**) Representative flow cytometry plots showing CD4^+^, CD8^+^, granzyme B^+^, and IFN-γ^+^CD8^+^ T cells among CD45^+^ tumor-infiltrating cells (*n* = 5). (**E**–**H**) Quantification of CD8^+^ T cells (**E**), CD4^+^ T cells (**F**), IFN-γ^+^CD8^+^ T cells (**G**), and granzyme B^+^CD8^+^ T cells (**H**) within the CD45^+^ cell population. (**I**–**K**) MB49 cells were implanted subcutaneously into the right flanks of C57BL/6J mice (*n* = 5). Mice were treated with TM (20 mg/kg), PD-1 antibody (50 μg per mouse), IgG2a isotype control (50 μg per mouse), or a combination via intraperitoneal injection on day 7. Tumor growth (**I**), tumor images (**J**), and tumor weight (**K**) are shown. (**L**) Representative flow cytometry plots showing CD4^+^, CD8^+^, granzyme B^+^ (GB), and IFN-γ^+^CD8^+^ T cells among CD45^+^ tumor-infiltrating cells (*n* = 5). (**M**–**O**) Quantification of CD8^+^ T cells (**M**), IFN-γ^+^CD8^+^ T cells (**N**), and granzyme B^+^CD8^+^ T cells (**O**) within the CD45^+^ cell population. Scale bars: 1 cm. Data are shown as mean ± SD. Statistical significance was determined using 1-way ANOVA. *P < 0.05; **P < 0.01; ***P < 0.001.

Building upon our earlier findings that CRISPR-mediated deletion of SIRT2 suppresses tumor growth and immune evasion via PD-L1 downregulation, we hypothesized that pharmacologic inhibition of SIRT2 could synergize with PD-1 immune checkpoint blockade (ICB) to enhance the efficacy of immunotherapy in bladder cancer. To test this, we treated mice bearing MB49 bladder tumors with either the SIRT2 inhibitor AGK2, anti–PD-1 antibody, or a combination of both agents. While monotherapy with either AGK2 or anti–PD-1 significantly inhibited tumor growth, their combination led to a dramatic and synergistic suppression of tumor progression. Compared with the IgG2a isotype control, mice treated with the combined AGK2 and anti–PD-1 therapy enhanced antitumor immune responses (Figure 8, A–C). Flow cytometry analysis revealed a significant increase in the infiltration of CD8^+^, but not CD4^+^, T cells in tumors from the combination group, exceeding the levels observed in either monotherapy group (Figure 8, D–F). Moreover, CD8^+^ T cells from the combination-treated tumors displayed enhanced effector function, as indicated by increased expression of IFN-γ and granzyme B (Figure 8, D, G, and H). These results suggest that the enhanced antitumor immunity observed with combined SIRT2 inhibition and ICB is primarily driven by reinvigoration of CD8^+^ cytotoxic T cell activity.

AGK2 has been reported to exhibit only modest selectivity over SIRT1 and SIRT3, whereas comparative studies indicate that TM is among the most selective SIRT2 inhibitors (51, 52). However, unlike SIRT2, CRISPR-targeted deletion of SIRT1 or SIRT3 did not affect USP22 protein expression levels (Supplemental Figure 6). Consistent with our observations in SIRT2-KO tumor cells, treatment with a more specific SIRT2 inhibitor, TM, suppressed both PD-L1 and USP22 expression (Supplemental Figure 5). These results indicate that specific SIRT2 inhibition enhanced antitumor immunity. To support this, we further demonstrated that in vivo administration of TM to tumor-bearing mice significantly inhibited tumor growth (Figure 8, I–K). Immunophenotypic analysis revealed a robust increase in intratumoral CD8^+^ T cell infiltration, accompanied by enhanced production of IFN-γ, granzyme B, and TNF-α, indicating potentiation of antitumor effector function (Figure 8, L–O). Collectively, these findings demonstrate that SIRT2 inhibition boosts CD8^+^ T cell antitumor immunity, at least in part through downregulation of PD-L1 expression.

### Combined SIRT2 and USP22 inhibition exhibits potent antitumor immunity.

**Figure 9:**
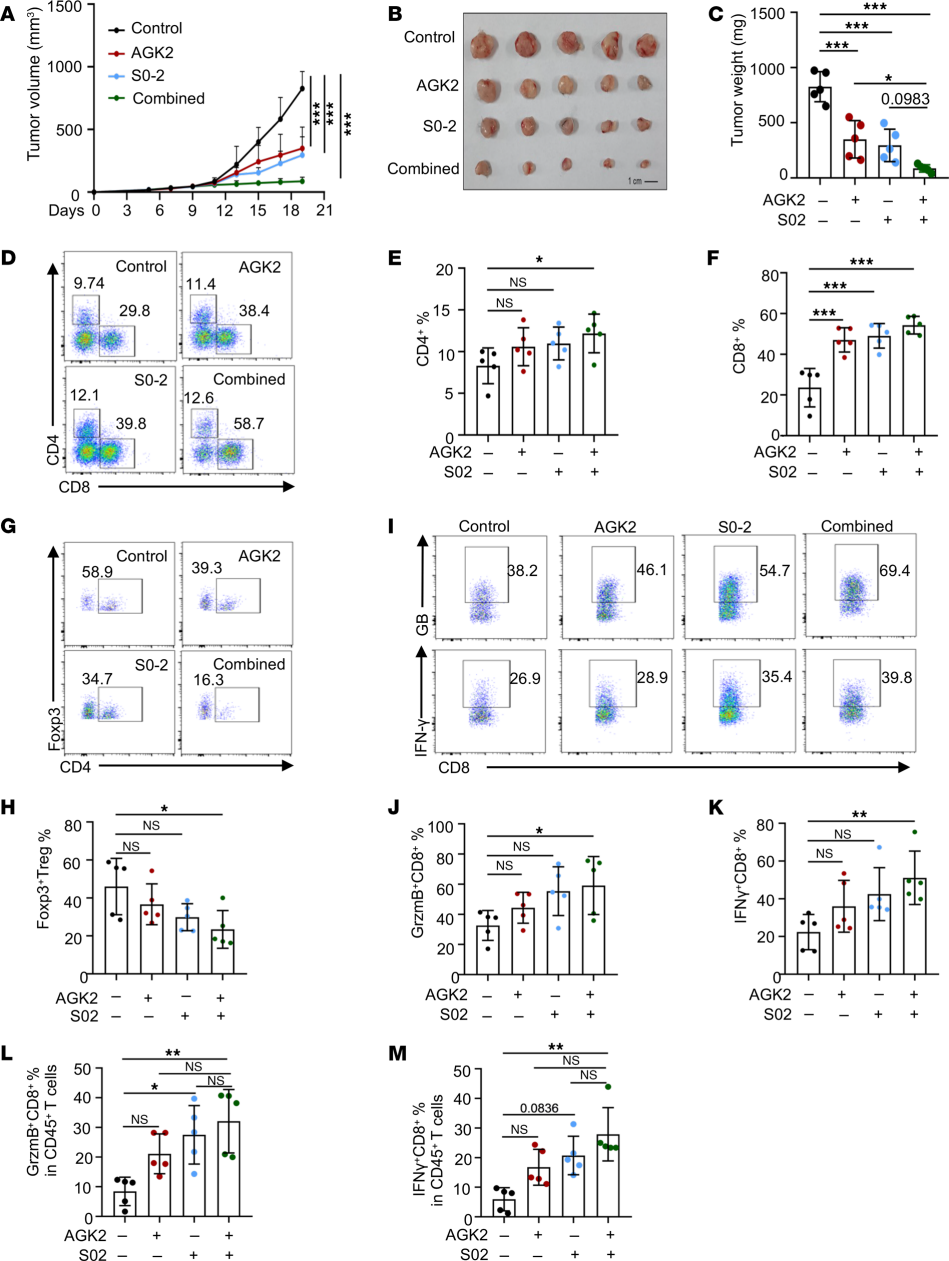
Combined SIR2 and USP22 inhibition in cancer treatment. (**A**–**C**) MB49 cells were implanted subcutaneously into the right flanks of C57BL/6J mice (*n* = 5). Mice were treated with AGK2 (10 mg/kg), USP22i-S02 (20 mg/kg), or a combination via intraperitoneal injection on day 7. Tumor growth (**A**), tumor images (**B**), and tumor weight (**C**) are shown. Scale bar: 1 cm. (**D**–**F**) Tumor-infiltrated CD4^+^ and CD8^+^ T cells were analyzed by flow cytometry. Representative flow cytometry plots showing CD4^+^ and CD8^+^ T cells (**D**) and data for 5 mice from each group (**E** and **F**) are shown. (**G** and **H**) CD4^+^FoxP3^+^ Tregs in gated CD4^+^ T cells were analyzed. (**I**–**M**) Granzyme B^+^ and IFN-γ^+^CD8^+^ T cells were determined by intracellular staining. Representative images (**I**) and granzyme B^+^ and IFN-γ^+^ frequency in CD8^+^ T cells (**J** and **K**) or CD45^+^ cells (**L** and **M**) are shown. Data are shown as mean ± SD. Statistical significance was determined using 1-way ANOVA. *P < 0.05; **P < 0.01; ***P < 0.001.

Given that USP22 and SIRT2 exert both overlapping and distinct tumor-promoting functions, there is a strong rationale for their combined inhibition to enhance antitumor immunity. Consistent with this concept, inhibition of either USP22 or SIRT2 alone significantly suppressed tumor growth, and dual targeting produced a greater effect. Specifically, cotreatment with the USP22 inhibitor S02 and the SIRT2 inhibitor AGK2 in established tumor models led to near-complete tumor suppression, supporting a cooperative, and potentially synergistic, interaction (Figure 9, A–C).

Further immune profiling revealed an increase in CD8^+^ T cell infiltration following treatment with either USP22i-S02 or AGK2, with the combination producing the most pronounced effect. In contrast, CD4^+^ T cell infiltration remained largely unchanged (Figure 9, D–F). Consistent with our recent findings, USP22 inhibition significantly reduced intratumoral Treg populations, an effect that was further enhanced by cotreatment with the SIRT2 inhibitor AGK2 (Figure 9, G and H). Functional analyses demonstrated a robust enhancement of CD8^+^ T cell effector activity by either USP22i-SO2 or AKG2 treatment, as evidenced by increased production of IFN-γ and granzyme B, and this efficacy was further enhanced by their combination (Figure 9, I–M). Collectively, these results indicate that combined treatment with S02 and AGK2 potentiates intratumoral antitumor immunity in tumor-bearing mice in a clinically relevant setting.

### Positive correlation of SIRT2 with USP22 and PD-L1 expression in human cancers.

**Figure 10:**
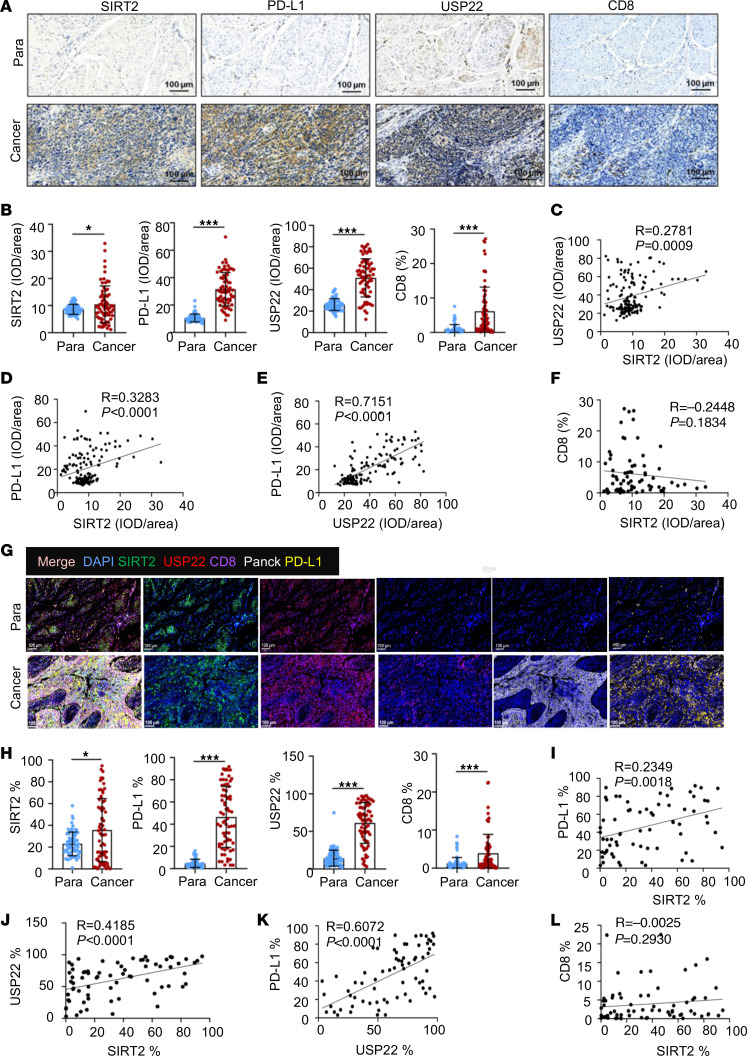
Positive correlation of SIRT2 with PD-L1 expression in human bladder cancer. A TMA with 70 bladder cancer sections with control adjacent normal tissue was used for the staining. (**A**) Representative IHC staining images of SIRT2, PD-L1, USP22, and CD8 in human bladder carcinoma and adjacent para-cancerous tissues. Scale bars: 100 μm. (**B**–**F**) Quantification of staining intensities for SIRT2, PD-L1, USP22, and CD8 in bladder carcinoma and adjacent tissues (*n* = 70). Correlation analysis of SIRT2 with PD-L1 (**D**) and USP22 (**C**), USP22 with PD-L1 (**E**), and SIRT2 with CD8 (**F**) protein levels in bladder carcinoma (*n* = 70). (**G**) Representative immunofluorescence staining images of SIRT2, PD-L1, USP22, and CD8 in human bladder carcinoma and adjacent para-cancerous tissues. Scale bars: 100 μm. (**H**) Quantification of staining intensities for SIRT2, PD-L1, USP22, and CD8 in bladder carcinoma and adjacent tissues (*n* = 70). (**I**–**L**) Correlation analysis of SIRT2 with PD-L1 (**I**) and USP22 (**J**), USP22 with PD-L1 (**K**), and SIRT2 with CD8 (**L**) protein levels in bladder carcinoma (*n* = 70). Data are shown as mean ± SD. Statistical significance was determined using an unpaired 2-tailed Student’s *t* test for **B** and **H** or Pearson’s correlation analysis for **C**–**F** and **I**–**L**. *P < 0.05; **P < 0.01; ***P < 0.001.

Our data clearly demonstrate that SIRT2 enhances PD-L1 expression by deacetylating USP22 to promote USP22 self-deubiquitination. To evaluate the clinical relevance of these findings in human cancers, we performed IHC analysis of SIRT2, USP22, and PD-L1 protein expression, as well as CD8^+^ T cell infiltration in 74 bladder carcinomas, with adjacent nontumorous tissues as controls. Quantitative analysis revealed significantly elevated levels of SIRT2, USP22, and PD-L1 proteins in tumor tissues compared with adjacent nontumorous (para-cancerous) tissues (Figure 10, A and B). Consistent with our mechanistic findings, SIRT2 expression showed a strong positive correlation with both USP22 and PD-L1 levels in bladder carcinoma (Figure 10, C and D). Furthermore, USP22 and PD-L1 expression were also positively correlated in both cancer types (Figure 10E). In contrast, analysis of The Cancer Genome Atlas (TCGA) database did not show their positive correlation at mRNA levels (Supplemental Figure 13), further supporting our observations that SIRT2 regulates both USP22 and PD-L1 at the posttranscriptional level.

In addition, analysis of a melanoma tissue microarray (TMA) comprising 70 patient samples revealed elevated expression of SIRT2, USP22, and PD-L1, with strong positive correlations among all 3 markers (Supplemental Figure 14). These findings further support the notion that the SIRT2/USP22/PD-L1 axis represents a potentially conserved mechanism of tumor immune evasion across cancer types.

Given our data indicating that SIRT2 promotes PD-L1 expression, higher SIRT2 levels would be expected to associate with diminished CD8^+^ T cell–mediated antitumor immunity. However, the inverse correlation between SIRT2 expression and CD8^+^ T cell infiltration was modest and did not reach statistical significance by IHC (Figure 10F). Recognizing the limited sensitivity of IHC for detecting low-frequency CD8^+^ T cells, we further performed immunofluorescence staining. This analysis confirmed elevated expression of SIRT2, USP22, and PD-L1 in tumor tissues, with significant positive correlations among these markers (Figure 10, G–K). Consistently, the negative correlation between SIRT2 expression and CD8^+^ T cell infiltration remained weak and did not reach statistical significance (Figure 10L).

In summary, our study uncovers a regulatory pathway in which SIRT2-mediated deacetylation of USP22 prevents its self-ubiquitination, thereby upregulating PD-L1 expression. These findings not only deepen our understanding of PD-L1 regulation but also suggest that targeting SIRT2 in combination with PD-1/PD-L1 ICB represents a promising therapeutic strategy to enhance antitumor efficacy (Supplemental Figure 15).

## Discussion

Through an unbiased screening approach, we identified SIRT2 as a positive regulator of tumor PD-L1 protein expression, uncovering a previously unrecognized molecular mechanism by which SIRT2 modulates the catalytic activity of the cancer stem cell gene USP22 to promote tumor immune evasion. This conclusion is supported by the following findings: (a) inhibition of SIRT2, and to a lesser extent SIRT3, but not other sirtuin family members, significantly reduces PD-L1 protein levels in tumor cells without affecting its mRNA expression, indicating a posttranscriptional mode of regulation. (b) SIRT2 inhibition also led to a specific reduction in USP22 protein levels, and reconstitution of USP22 in SIRT2-deficient tumor cells fully restored PD-L1 protein expression, implicating USP22 as a critical mediator in this pathway. (c) Biochemical analyses confirmed that USP22 is a direct substrate of SIRT2 deacetylase activity; SIRT2-mediated deacetylation enhances USP22’s auto-deubiquitination, thereby stabilizing the protein. (d) Acetylation of key lysine residues within the catalytic core of USP22 inhibits its enzymatic function, impairing its ability to deubiquitinate itself and PD-L1. (e) Pharmacological inhibition of SIRT2 synergizes with ICB to suppress tumor progression, highlighting its therapeutic potential. (f) IHC analyses of human bladder cancer and melanoma samples revealed strong positive correlations among SIRT2, USP22, and PD-L1 at the protein level; however, these correlations were not observed at the mRNA level in TCGA datasets, further supporting the role of posttranscriptional regulation in this axis.

PD-1/PD-L1 ICB monotherapy has limited clinical efficacy in bladder cancer (3–5). In addition to the minimal benefit, most patients are exposed to substantial ICB treatment–related toxicities (8–11). Therefore, there is an urgent need for combination strategies that enhance immune response while minimizing resistance. Our findings provide a strong rationale for combining SIRT2 inhibition with ICB. Genetic ablation or pharmacological inhibition of SIRT2 not only decreased PD-L1 expression in tumor cells but also enhanced CD8^+^ T cell infiltration, activation, and survival, leading to improved antitumor immune responses in vivo. Importantly, treatment with the SIRT2 inhibitor AGK2 significantly synergized with anti–PD-1 antibodies to suppress tumor growth more effectively than either treatment alone, accompanied by increased intratumoral cytotoxic T cell activity. However, AGK2 exhibits only modest selectivity over SIRT1 and SIRT3, and a direct comparison indicated that TM is the most specific SIRT2 inhibitor (51, 52). It has been shown that targeting SIRT2 with inhibitors like AGK2 may offer a promising approach for cancer treatment (51, 53, 54). SIRT2 is a protein deacetylase that can play a role in promoting tumor growth by regulating oncogenic factors like c-Myc (51). By contrast, SIRT2 inhibition destabilizes fibrinogen-like protein 1 (53) and MutL protein homolog 1 (54) to enhance immunotherapeutic efficacy. Therefore, SIRT2 is a multifunctional mechanistic regulator of immune evasion and a tractable therapeutic target with both onco-targeting and immune-boosting efficacies for cancer treatment.

Our study revealed that SIRT2 promotes tumor PD-L1 expression primarily through the deubiquitinating enzyme USP22, a known regulator of PD-L1 stability (38, 55). Although several deubiquitinating enzymes have been implicated in PD-L1 regulation, including USP5, USP7, USP8, USP9X, USP20, USP22, and CSN5 (31–34, 37–39), our data suggest that SIRT2 selectively modulates PD-L1 expression via USP22. Specifically, targeted deletion of SIRT2 led to a pronounced reduction in USP22 protein levels, while other PD-L1–associated deubiquitinases remained largely unaffected. Notably, reexpression of USP22 in SIRT2-deficient cancer cells fully restored PD-L1 protein levels, strongly indicating that USP22 acts downstream of SIRT2 in this regulatory axis. These findings support a model in which SIRT2 promotes PD-L1 accumulation by stabilizing USP22, which in turn deubiquitinates and protects PD-L1 from proteasomal degradation. Beyond its role in PD-L1 regulation, USP22 has emerged as a multifaceted oncogenic factor in cancer biology. Recent studies have shown that USP22 contributes to tumor immune evasion by enhancing the immunosuppressive activity of Tregs (56–58). Additionally, USP22 is known to drive oncogenesis by modulating key pathways that inhibit tumor suppressors such as p53 and Myc, regulating cyclin stability, maintaining cancer stem cell properties, and supporting metabolic reprogramming in tumor cells (59–61). Given these broad oncogenic roles, our findings offer a compelling rationale for targeting the SIRT2/USP22 axis as a therapeutic strategy. Dual inhibition of SIRT2 and USP22 could not only suppress PD-L1–mediated immune escape but also disrupt multiple tumor-promoting pathways, providing a multilayered therapeutic synergy that may enhance the efficacy of current immunotherapies and reduce resistance in cancer treatment.

One unexpected and novel finding of our study is that the deubiquitinase activity of USP22 is regulated by SIRT2-mediated deacetylation. Posttranslational modifications have emerged as critical regulatory mechanisms that fine-tune enzymatic functions, including stability, localization, and catalytic activity. Acetylation is well documented to enhance the activity of certain kinases, for example, promoting MEK1 activity and downstream ERK signaling (62). However, the role of acetylation in modulating the enzymatic activity of deubiquitinases has remained largely unexplored. Our unbiased proteomic analysis identified 2 lysine residues located within the core catalytic peptidase domain of USP22 that undergo acetylation. Site-directed mutagenesis of these lysines to arginine, which mimics the deacetylated state, significantly enhanced USP22’s deubiquitination activity toward both itself and its substrate PD-L1. In contrast, substitution of the same residues with glutamine, mimicking constitutive acetylation, almost completely abolished USP22’s enzymatic function. Although the precise molecular mechanisms by which acetylation impairs USP22 activity are not fully elucidated, we employed MD simulations and MM/PBSA binding free energy calculations to investigate the structural basis of this regulation. These simulations revealed that the nonacetylated form of USP22 forms a stable complex with ubiquitin, adopting a low-energy conformation within a single energy well. This suggests a strong and stable interaction essential for catalytic efficiency. In contrast, the acetylated form displayed higher structural flexibility and reduced binding affinity for ubiquitin, consistent with diminished enzymatic activity. In addition, it is possible that this acetylation may also affect USP22 crosstalk with its E3 ubiquitin ligase; however, the E3 ubiquitin ligase responsible for USP22 ubiquitination has not yet been identified. Nevertheless, these findings uncover a previously unrecognized layer of regulation in which SIRT2-mediated deacetylation governs the catalytic competency of USP22, adding important insight into the broader role of posttranslational modifications in modulating deubiquitinase function and immune checkpoint regulation.

Over the last decade, we and others have demonstrated that USP22 functions as an oncogene by inhibiting cell apoptosis and promoting cell cycle progression (63–69). USP22 is essential to maintain triple-negative breast cancer (TNBC) stemness through regulating integrin expression, and genetic and pharmacological inhibition of USP22 blocked TNBC growth and lung metastasis (59, 70). More recently, we have demonstrated that USP22 is a FoxP3-specific deubiquitinase, and its deletion partially diminishes Treg suppressive functions. Mice with Treg-specific USP22 deletion exhibited vigorous tumor rejection (56, 58). USP22 was also shown to inhibit antitumor immunity through upregulation of PD-L1 and CD73 (38, 55), 2 checkpoint receptors responsible for evading antitumor immunosurveillance. Therefore, it will be interesting for future studies to test whether simultaneous targeting of SIRT2 and USP22 synergizes with ICB achiever superior antitumor therapeutic efficacy in treatment of most, if not all, human malignancies.

Although our mechanistic studies demonstrate that SIRT2 promotes PD-L1 expression and is therefore expected to suppress CD8^+^ T cell–mediated antitumor immunity, analysis of a bladder cancer TMA (*n* = 74) revealed only a modest, nonsignificant inverse correlation between SIRT2 expression and CD8^+^ T cell infiltration. This finding was consistent across both IHC and immunofluorescence analyses, suggesting that the lack of statistical significance is unlikely to be solely due to technical limitations in detection sensitivity. One plausible explanation is that these TMA specimens were derived from surgical samples, many of which may have been exposed to prior therapies that alter immune composition within the tumor microenvironment. In addition, CD8^+^ T cell infiltration is governed by multiple factors beyond PD-L1, including chemokine gradients, stromal barriers, and broader immunosuppressive networks, which may obscure a direct correlation with SIRT2 expression in human samples. Future studies using treatment-naive tumor cohorts, coupled with higher-resolution approaches such as spatial transcriptomics or single-cell profiling, are needed to more precisely define the relationship between the SIRT2/USP22/PD-L1 axis and CD8^+^ T cell dynamics in vivo.

## Methods

### Sex as a biological variable.

All mouse experiments were double-blind. Both male and female age-matched mice were used.

### Animal studies.

C57BL/6J mice and BALB/c nude mice (6–8 weeks old) were purchased from the Animal Center of Dalian Medical University. For the subcutaneous tumor model, 1 × 10^6^ cells were injected into the right flank of C57BL/6J or BALB/c nude mice. Tumor size was measured every 2 days starting from day 5 after implantation using a caliper, and tumor volume was calculated using the formula: volume = length × width^2^/2.

For combination treatment experiments, tumor-bearing mice were randomized into treatment or control groups. Treatments included AGK2 (10 mg/kg, TargetMol, T6371), TM (75 mg/kg, TargetMol, T3447), USP22 inhibitor S0-2 (20 mg/kg), PD-1 antibody (50 μg, Bio X Cell, BE0273), rat IgG2a isotype control (50 μg, Bio X Cell, BE0089), or a combination, administered via intraperitoneal injection starting on day 7 (8 injections total). Mice were euthanized when tumors reached 2,000 mm^3^ or showed ulceration.

### Cell culture, transfection, generation of stable cell lines, and cell treatment.

Human bladder cancer cell line T24, murine bladder cancer cell line MB49, and human endometrial cancer cell line KLE were purchased from Procell Life Science & Technology. Human melanoma cell line A375, murine melanoma cell line B16, and HEK293T cells were obtained from ATCC. MB49, A375, B16, and HEK293T cells were cultured in DMEM with 10% FBS. T24 cells were cultured in McCoy’s 5A medium with 10% FBS. Cells were maintained at 37°C in a humidified incubator with 5% CO_2_. Transfections were performed using Lipofectamine 3000 (Invitrogen, L3000150) according to the manufacturer’s instructions, and cells were harvested 48 hours after transfection.

CRISPR/Cas9-mediated SIRT2-KO, SIRT1-KO, SIRT3-KO, and PD-L1 stable overexpression lines were generated using Cas9 ribonucleoprotein complexes and lentiviral packaging, performed by Beijing Syngentech Co., Ltd. CRISPR/Cas9-mediated USP22 stable overexpression line was generated using Cas9 ribonucleoprotein complexes and lentiviral packaging, performed by Miaoling Biology. SIRT2-KO, SIRT1-KO, and SIRT3-KO cells were selected with puromycin (MedChemExpress, HY-B1743Aa). PD-L1– and USP22-overexpressing cells were selected with G418 (MedChemExpress, HY-17561) for at least 3 days. For protein degradation assays, transfected HEK293T or T24 cells were treated with cycloheximide (CHX) (Cell Signaling Technology, 2112) at indicated time points. T24 cells were treated with 20 μM AGK2, 20 μM TM, or 20 μM SirReal (TargetMol, T6984) as specified. For protein degradation pathway analysis, SIRT2-KO T24 cells were treated with the proteasome inhibitor MG132 (MedChemExpress, HY-13259) or the lysosomal inhibitor NH_4_Cl (MedChemExpress, HY-Y1269).

### siRNA and plasmids.

All siRNAs were synthesized by Sangon Biotech. Sequences are provided in Supplemental Table 1. HA-ubiquitin, His-ubiquitin, HA-USP22, Myc-USP22, Myc-C185A-USP22, and Myc-PD-L1 plasmids were previously described (25, 26). Myc-K59R, K88R, K243R, K382R, K505R, and KR-USP22 mutants and Flag-SIRT2, Flag-H187Y-SIRT2, and pcDNA3.1 plasmids were purchased from Miaoling Biology. The siRNA and CRISPR guide sequences are listed in Supplemental Table 4.

### Flow cytometry analysis of membrane PD-L1.

All antibodies used in this study are listed in Supplemental Table 3. Cells were washed twice with PBS, harvested using Accutase (Thermo Fisher Scientific, 00-4555-56, and centrifuged at 300*g* for 5 minutes. Cells were incubated with fixable viability dye (Invitrogen, L34955) and stained with APC-conjugated anti-human PD-L1 (Thermo Fisher Scientific, 17-5983-42) or anti-mouse PD-L1 (BD Biosciences, 564715) for 30 minutes at 4°C. After washing and resuspension in FACS buffer, PD-L1 expression was analyzed using a flow cytometer (Thermo Fisher Scientific). Data were processed with FlowJo (BD Biosciences) and GraphPad Prism software.

### Tumor-infiltrating lymphocyte analysis.

Mice were euthanized by cervical dislocation, and tumors were excised, weighed, and dissociated in collagenase IV (4 mg/mL, Worthington, LS004188) and DNase I (Solarbio, D8071) at 37°C. Single-cell suspensions were prepared using a 70 μm strainer. Fc receptors were blocked with anti-CD16/32 (BD Biosciences, 553141). For surface staining, cells were labeled with viability dye (Invitrogen, L34955), Alexa Fluor 700–CD45 (BioLegend, 147716), FITC–CD4 (BD Biosciences, 553046), BV510–CD8 (BD Biosciences, 563068), BB700–PD-1 (BD Biosciences, 566514), BV711–TIM-3 (BD Biosciences, 747622), BV421–LAG-3 (BD Biosciences, 740072), and APC–PD-L1 for 30 minutes at 4°C.

For intracellular staining, cells were fixed and permeabilized using BD Transcription Factor Buffer Set (BD Biosciences, 562574), then stained with APC-FoxP3 (Thermo Fisher Scientific, 17-5773-82), RB780-TOX (BD Biosciences, 570192), and Alexa Fluor 647-NR4A1 (BD Biosciences, 566735). Cytokine staining involved stimulation with Leukocyte Activation Cocktail (BD Biosciences, 550583) followed by staining with PE-granzyme B (BioLegend, 372208), PE-Cy7-IFN-γ (BD Biosciences, 557649), and BV605-TNF-α (BioLegend, 506329). For apoptosis assays, cells were resuspended in annexin V binding buffer (BD Biosciences, 556547) and stained with FITC–annexin V. Samples were analyzed by flow cytometry and processed using FlowJo and GraphPad.

### Co-IP and Western blotting.

Cells were lysed in RIPA buffer (Beyotime, P0013C) with protease inhibitors. Lysates were incubated on ice for 30 minutes and centrifuged at 12,000*g* for 10 minutes at 4°C. Supernatants were precleared with Protein G Sepharose (GE Healthcare, 17-0618-02), then immunoprecipitated with the indicated antibodies overnight. Protein A/G beads were added for 2 hours, and complexes were washed, eluted in loading buffer, and boiled. Proteins were separated by SDS-PAGE (8%–10%) and transferred to nitrocellulose membranes. Membranes were blocked with 5% milk in TBST for 2 hours, incubated with primary antibodies overnight at 4°C, washed, and incubated with HRP-conjugated secondary antibodies. Signals were detected using ECL substrate (Abbkine, BMU102-CN) and quantified with Bio-Rad imaging software. Membranes were stripped (Solarbio, SW3020) and reprobed as needed. Antibodies used are listed in Supplemental Table 2.

### In vitro deacetylation assay.

HEK293T cells were transiently transfected with His-USP22. Twenty-four hours later, the cells were treated with 20 mM nicotinamide (Proteintech, 98-92-0) for 10 hours to hyperacetylate proteins. Subsequently, the cells were collected and lysed with the IP buffer. Proteins were purified using Dynabeads (Cytiva, 17371201). For in vitro deacetylation assay, the SIRT2 enzyme (R&D, 4358-DA-050), purified His-USP22 protein as a substrate, and NAD (Proteintech, 53-84-9) as a cofactor to activate the SIRT2 were mixed within a tube. After incubation at 37°C for 3 hours, the samples were run on 10% SDS-PAGE.

### RT-qPCR.

Total RNA was extracted using the Total RNA Kit I (Omega, R6834-01) and reverse-transcribed using the iScript cDNA Synthesis Kit (Bio-Rad, 1708891). qPCR was performed with the SYBR Green Premix Pro Taq HS qPCR Kit (Accurate Biotechnology, AG11701) on a CFX96 system (Bio-Rad). Relative expression was calculated using the ΔCt method and normalized to β-actin. Primers were obtained from Thermo Fisher Scientific and are listed in Supplemental Table 5.

### MS analysis.

To identify USP22 acetylation sites, Myc-USP22 and Flag-SIRT2 or control plasmids were cotransfected into HEK293T cells for 48 hours. Lysates were immunoprecipitated with anti-Myc antibody, separated by SDS-PAGE, and subjected to MS analysis, performed by Amogene Biotech.

### Cell proliferation assays.

MB49 cell proliferation was measured using the CCK-8 kit (Absin, abs50003). Cells were seeded at 3 × 10^3^ cells per well in 96-well plates and incubated with CCK-8 reagent for 2 hours. Absorbance was measured at 450 nm.

### MD simulation.

Structures of USP22, ubiquitin, and SIRT2 were modeled using SwissModel. Templates included yeast UBP8 (PDB ID: 3MHS) for USP22 and human SIRT2 (PDB ID: 3ZGO). Acetylated USP22 models at K505 and K380 were generated using CHARMM-GUI (https://www.charmm-gui.org/). Protein-protein docking was conducted using ZDOCK (https://zdock.wenglab.org/). MD simulations were performed using GROMACS 5.1.5 (https://www.gromacs.org/). MM/PBSA was used to calculate binding free energies. Interaction diagrams were generated using LigPlot^+^ (https://www.ebi.ac.uk/thornton-srv/software/LigPlus). To identify the minimum energy states of the protein and protein-ligand complexes, Gibbs free energy landscapes were constructed using the gmx sham tool, with RMSD and Rg as the reaction coordinates.

The binding free energy was calculated using 

Δ*G*_binding_ = Δ*E*_MM_ + Δ*G*_solv_ − TΔ*S*_MM_, Δ*E*_MM_ = Δ*E*_int_ + Δ*E*_vdW_ + Δ*E*_elec_, and Δ*G*_solv_ = Δ*G*_PB_ + Δ*G*_SA_.

### IHC staining.

Human cancer tissue arrays containing 70 bladder cancer cases and 70 melanoma cases were purchased from AiFang Biological (AF-BlalSur2401 and AF-MelalSur2401). IHC staining was performed using an IHC kit according to the manufacturer’s protocol. Briefly, mouse tumor samples were fixed with 4% paraformaldehyde and embedded in paraffin. After paraffin embedding, paraffin sections were deparaffinized and rehydrated, and the antigen of tissues was repaired successively. The samples were blocked with goat serum blocking solution and incubated with primary antibodies at 4°C overnight. On the second day, the sections were incubated with HRP-conjugated secondary antibodies, stained with DAB substrate, and counterstained with hematoxylin. All images were acquired using a Nikon microscope and analyzed by Aipathwell software. The staining intensity was quantified by calculating the integrated optical density (IOD) per unit area. Primary antibodies in this study included SIRT2 (1:100, Proteintech, 66410-1-Ig), USP22 (1:200, Proteintech, 55110-1-AP), PD-L1 (1:500, Proteintech, 66248-1-Ig), CD8 (1:1,000, Servicebio, GB11068), and granzyme B (1:200; Proteintech, 13588-1-AP).

### Multiplexed immunofluorescence.

Human cancer tissue arrays containing 70 bladder cancer cases were purchased from AiFang Biological (AF-BlalSur2401). A 5-color multiplexed immunofluorescence panel was developed for simultaneous detection of SIRT2 (Proteintech, 66410-1-Ig), USP22 (Abcam, ab195289), PD-L1 (Huilanbio, abb5980), CD8 (Proteintech, 666868-1-Ig), PanCK (Huilanbio, HL32118), and nuclear counterstain DAPI (Huilanbio, RC05). Tyramide signal amplification was applied for sequential staining according to the manufacturer’s protocol with minor modifications. Briefly, TMA sections were deparaffinized, rehydrated, and subjected to antigen retrieval in Tris-EDTA buffer (pH 9.0) using a microwave oven (95°C, 20 minutes). Endogenous peroxidase was blocked with 3% H_2_O_2_ for 15 minutes, and nonspecific binding was blocked with 5% normal donkey serum for 30 minutes at room temperature (RT). For each staining cycle, sections were incubated with primary antibodies at 4°C overnight, followed by HRP-conjugated secondary antibody for 1 hour at RT and then tyramide working solution for 10 minutes at RT. Between cycles, antibody complexes were removed by microwave treatment in citrate buffer. After all cycles, nuclei were counterstained with DAPI for 8 minutes. Slides were mounted with antifade medium. All images were acquired using a multispectral scanner (Vectra Polaris, PerkinElmer) and analyzed by HALO software in a double-blind manner. SIRT2/USP22/PD-L1 expression was quantified specifically in PanCK^+^ tumor cells. Positive cell rate (%) = (positive tumor cells/total tumor cells) × 100%. CD8^+^ cell rate (%) = (positive cells/total DAPI^+^ cells) × 100%. At least 5 nonoverlapping fields (200 × 200 μm) per TMA core were analyzed, and the average value was used for statistical analysis.

### Statistics.

Statistical analyses were performed using GraphPad Prism 8. Data normality was assessed with the Shapiro-Wilk test. Differences between groups were analyzed using an unpaired 2-tailed Student’s *t* test or 1-way ANOVA. Pearson’s correlation was used to assess relationships between variables. Two-way ANOVA was used for comparisons of tumor growth and survival analysis. Data are shown as mean ± SD from at least 3 independent experiments. A *P* value < 0.05 was considered statistically significant.

### Study approval.

All animal care and experimental protocols for in vivo studies were approved by the Committee for the Care and Use of Laboratory Animals at Dalian Medical University (AEE24001).

### Data availability.

The authors confirm that data supporting the findings of this study are available in the article and supplemental materials when possible. Values for all data points found in graphs can be found in the Supporting Data Values file. All raw, uncropped Western blots are available in the supplemental materials. Additional details regarding data and protocols that support the findings of this study are available from the corresponding author upon request.

## Author contributions

NL performed the studies and analyzed the data. QG, HJ, GX, YZ, and SW conducted experiments. SM, ZZ, CS, BH, YL, WX, and ZL provided reagents. DDZ, PC, ZS, and DF designed the study and wrote the manuscript.

## Conflict of interest

DF is a cofounder of ExoMira Medicine Inc. and inventor of USP22-specific inhibitors.

## Funding support

This work is the result of NIH funding, in whole or in part, and is subject to the NIH Public Access Policy. Through acceptance of this federal funding, the NIH has been given a right to make the work publicly available in PubMed Central.

- NIH grants R01DK126908, R01DK120330, R01CA257520, and CA232347 to DF.
- National Natural Science Foundation of China (82073768) to ZS.
- Dalian High-Level Talent Innovation Support Program (2019RD03) to ZS.

## Supplementary Material

Supplemental data

Unedited blot and gel images

Supporting data values

## Version 1 06/02/2026

In-Press Preview

## Version 2 07/15/2026

Electronic publication
