# Supplementary material for: SIRT2-mediated deacetylation activates USP22 catalytic function for PD-L1 protein stabilization and tumor immune escape
Source: J Clin Invest. 2026 Jun 2;136(14):e198270. doi: 10.1172/JCI198270 (PMC13367968; doi:10.1172/JCI198270)
Supplement: Supplemental data [file jci-136-198270-s182.pdf]

SIRT2-Mediated Deacetylation Activates USP22 Catalytic Function for PD-L1 Protein Stabilization and Tumor Immune Escape

Supplemental Figures 1-15 and Tables 1-5

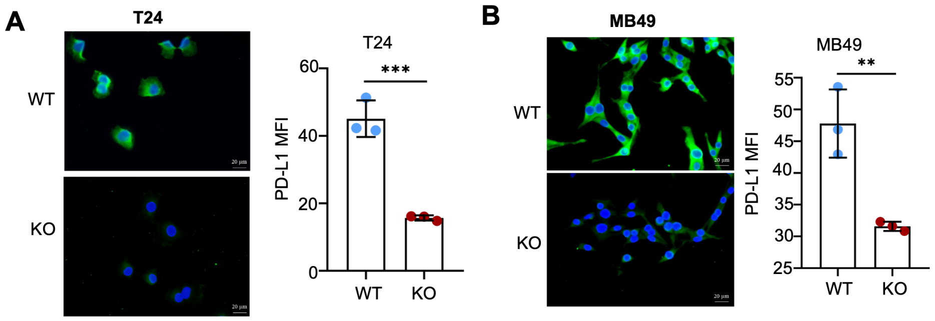

Fig. S1. Analysis of PD-L1 expression in multiple SIRT2-KO cells. WT and SIRT2 knockout (KO) human T24 **(A)** and mouse MB49 **(B)** bladder tumor cells were stained with anti–PD-L1 antibody followed by an Alexa Fluor 488–conjugated secondary antibody. Representative images and quantitative analysis from three independent experiments are shown. Statistical significance was determined by unpaired Student's *t* test (**P < 0.01; ***P < 0.001).

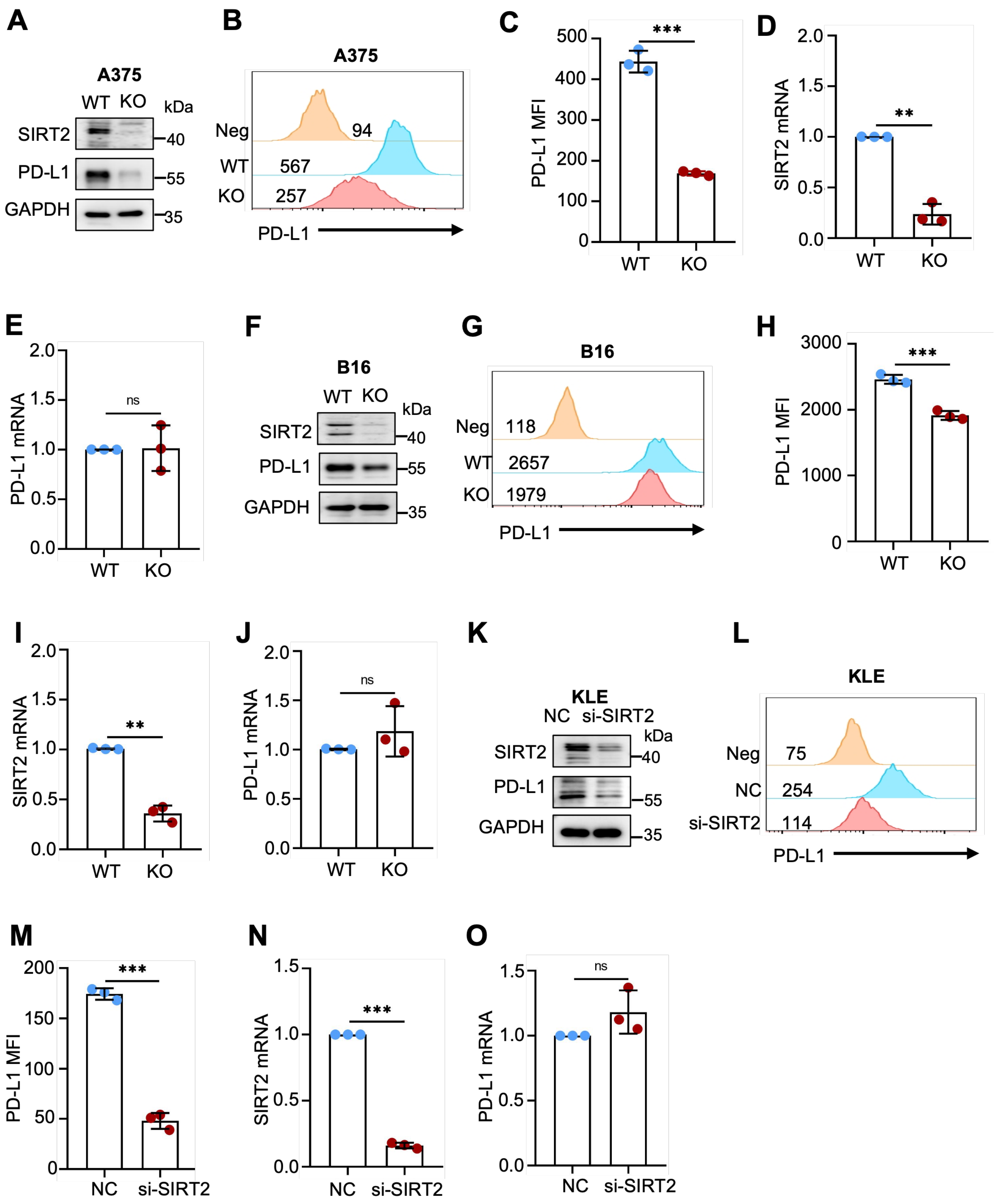

Fig. S2. Analysis of PD-L1 expression in multiple SIRT2-KO cells. (**A**) Western blotting analysis of SIRT2 and PD-L1 expression in WT A375 cells and SIRT2 KO A375 cells. (**B & C**) Representative flow cytometry plots and quantification of PD-L1 on the surface of WT and SIRT2 KO A375 cells. (**D & E**) qRT-PCR analysis of SIRT2 and PD-L1 mRNA levels in WT and SIRT2 KO A375 cells. (**F**) Western blotting analysis of SIRT2 and PD-L1 expression in WT and SIRT2 KO B16 cells. (**G & H**) Representative flow cytometry plots and quantification of PD-L1 on the surface of WT and SIRT2 KO B16 cells. (**I & J**) qRT-PCR analysis of SIRT2 and PD-L1 mRNA levels in WT and SIRT2 KO B16 cells. (**K**) Western blotting analysis of SIRT2 and PD-L1 expression in KLE cells transfected with SIRT2 siRNA. (**L & M**) Representative flow cytometry plots and quantification of PD-L1 on the surface of KLE cells transfected with SIRT2 siRNA. (**N & O**) qRT-PCR analysis of SIRT2 and PD-L1 mRNA levels in KLE cells transfected with SIRT2 siRNA. Statistical

significance was performed by two-tailed unpaired t test. *$P < 0.05$; **$P < 0.01$; ***$P < 0.001$; ns, not significant.

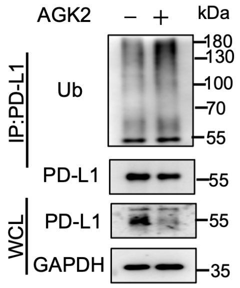

Fig. S3. The effect of pharmacological SIRT2 inhibition on PD-L1 ubiquitination in blader cancer cells. T24 cells were treated with or without SIRT2 inhibitor AGK2 and ubiquitination of PD-L1 was determined.

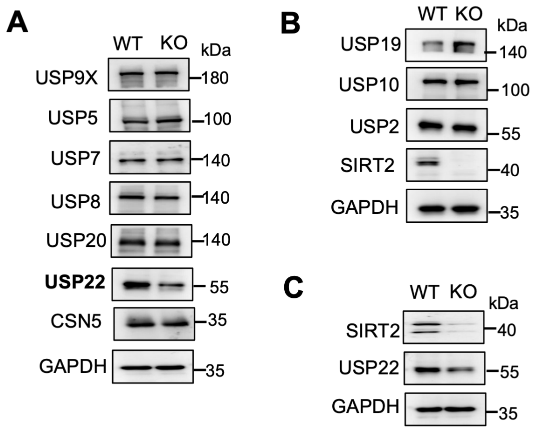

Fig. S4. Analysis of USP22 expression in targeted SIRT2 inhibition cells. Western blotting analysis of the indicated each deubiquitinase protein levels in SIRT2 KO A375 cells **(A)**, SIRT2 KO T24 cells **(B)** and B16 melanoma cells **(C).**

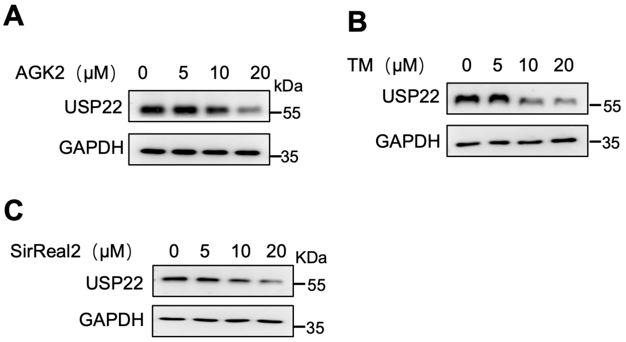

Fig. S5. The effect of pharmacological SIRT2 inhibition on USP22 expression. T24 cells were treated with SIRT2 inhibitors including AKG2 (A), TM (B) or SirReal2 (C) for 8 hours. Western blotting analysis of the indicated eachUSP22

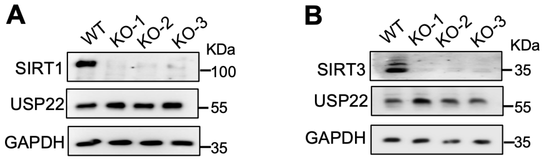

Fig. S6. Targeted Sirt1 and Sirt3 inhibition on USP22 expression. Sirt1 KO (A) and Sirt3 KO (B) T24 cells were generated by three independent CRISPR guides (KO1-3). USP22 protein expression levels were determined by western blotting with GAPDH as a loading control.

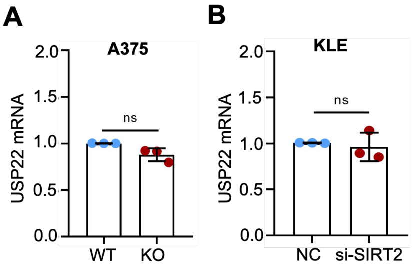

Fig. S7. Analysis of USP22 mRNA levels in USP22 KO A375 and KLE cells by qRT-PCR. Statistical significance was performed by two-tailed unpaired t test. ns. no significance.

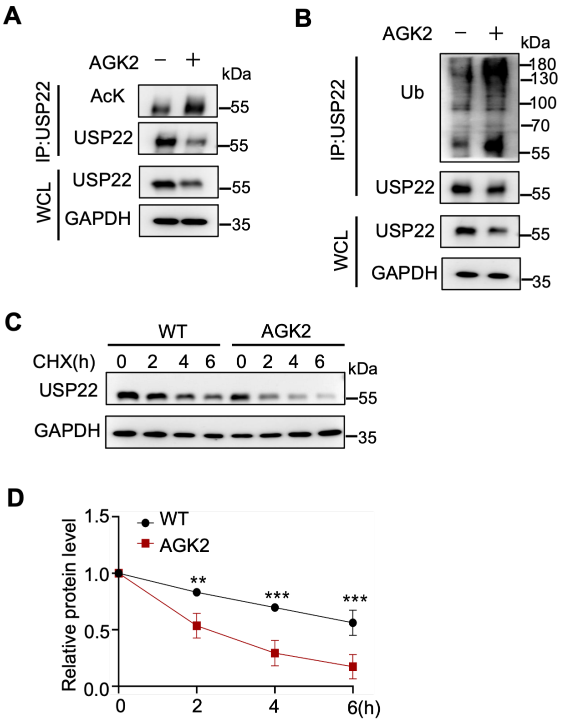

Fig. S8. Targeted SIRT2 suppression promoted USP22 degradation. (**A**) T24 cells treated with or without SIRT2 inhibitor AGK2 (20μM, 8h) were lysed and immunoprecipitated using anti-USP22 antibodies, followed by detected with anti-Pan-AC antibodies. (**B**) Western blotting and immunoprecipitation assay of USP22 ubiquitination in T24 cells treated with or without SIRT2 inhibitor AGK2. (**C & D**) T24 cells treated with or without AGK2 for the indicated times and the protein stability of USP22 were examined by Western blotting. Statistical significance was performed by two-tailed unpaired t test. $**P < 0.01$; $***P < 0.001$.

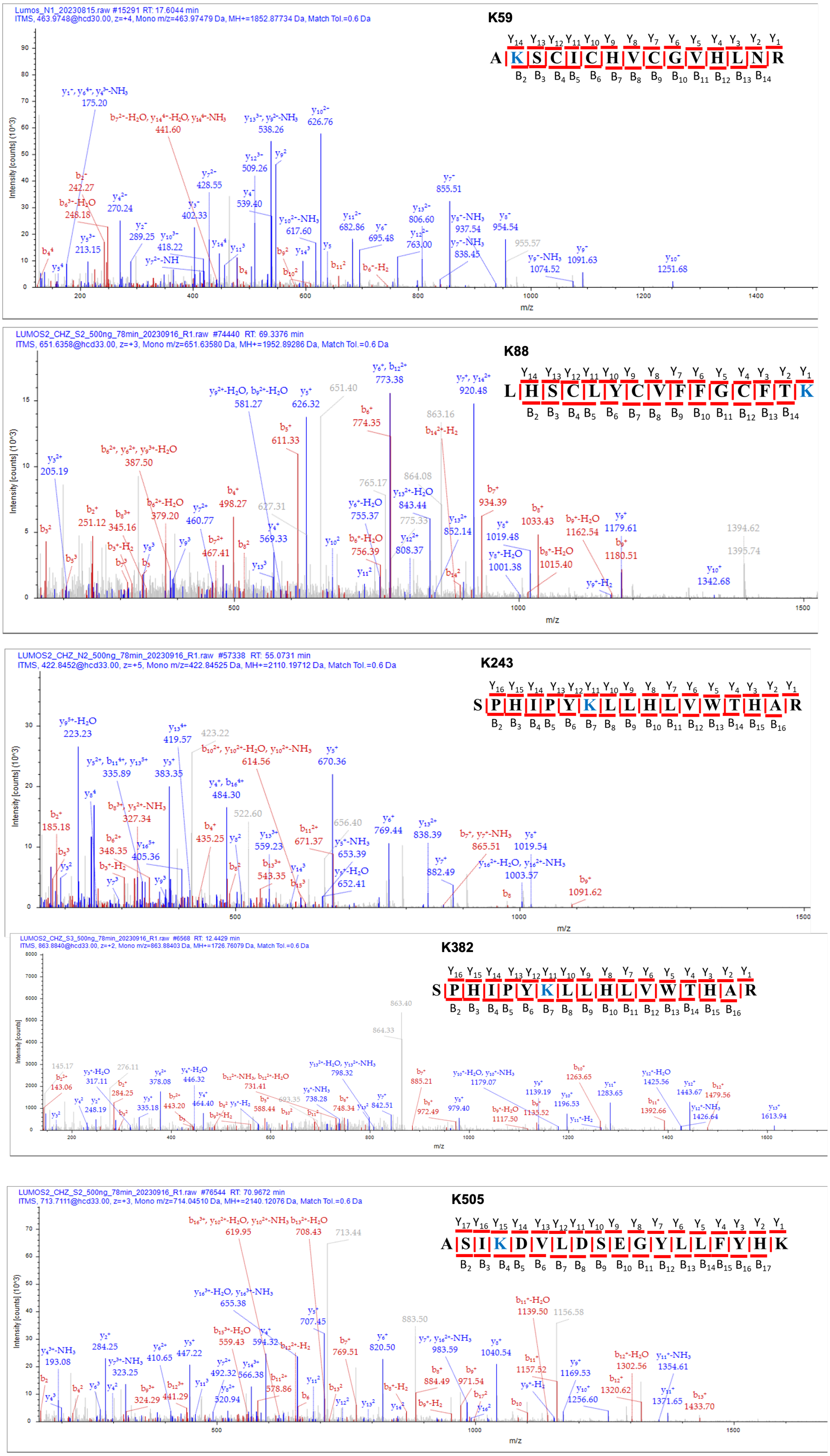

Fig. S9. Identification of USP22 acetylation residues using mass spectrometry analysis. The acetylated lysine residues at USP22 were highlighted in bold blue text.

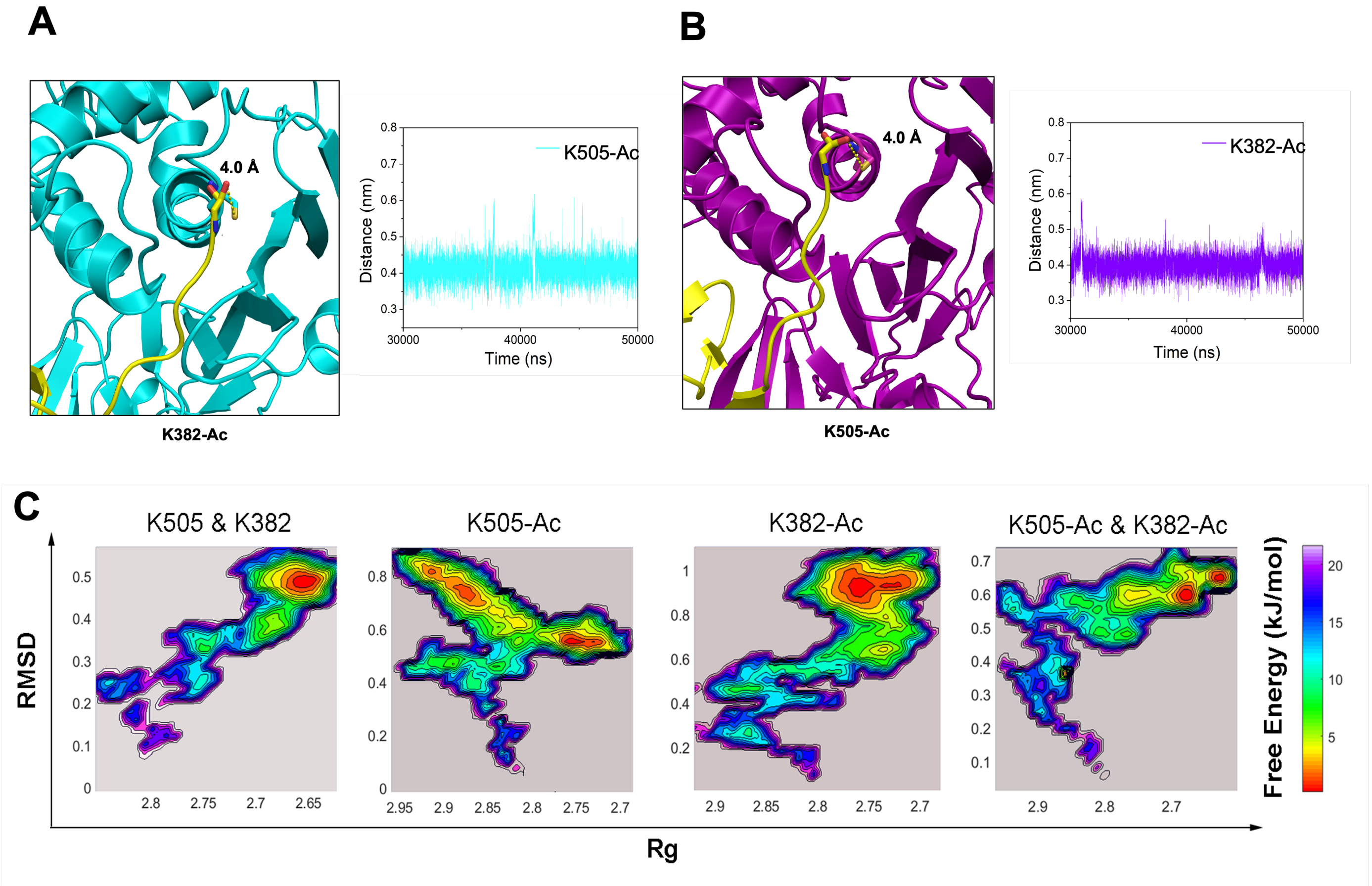

Fig. S10. The molecular dynamics (MD) simulations and MM/PBSA binding free energy calculations of the acetylated USP22 binding with ubiquitin. **(A, B)** Schematic diagram showing the ubiquitin-binding and acetylation sites of USP22 at lysine 382 (K382) (A) or 505 (K505) (B). **(C)** 50-ns molecular dynamics (MD) simulations for K505-acetylated, K382-acetylated, and double-acetylated (K505 and K382) UB–USP22 complexes.

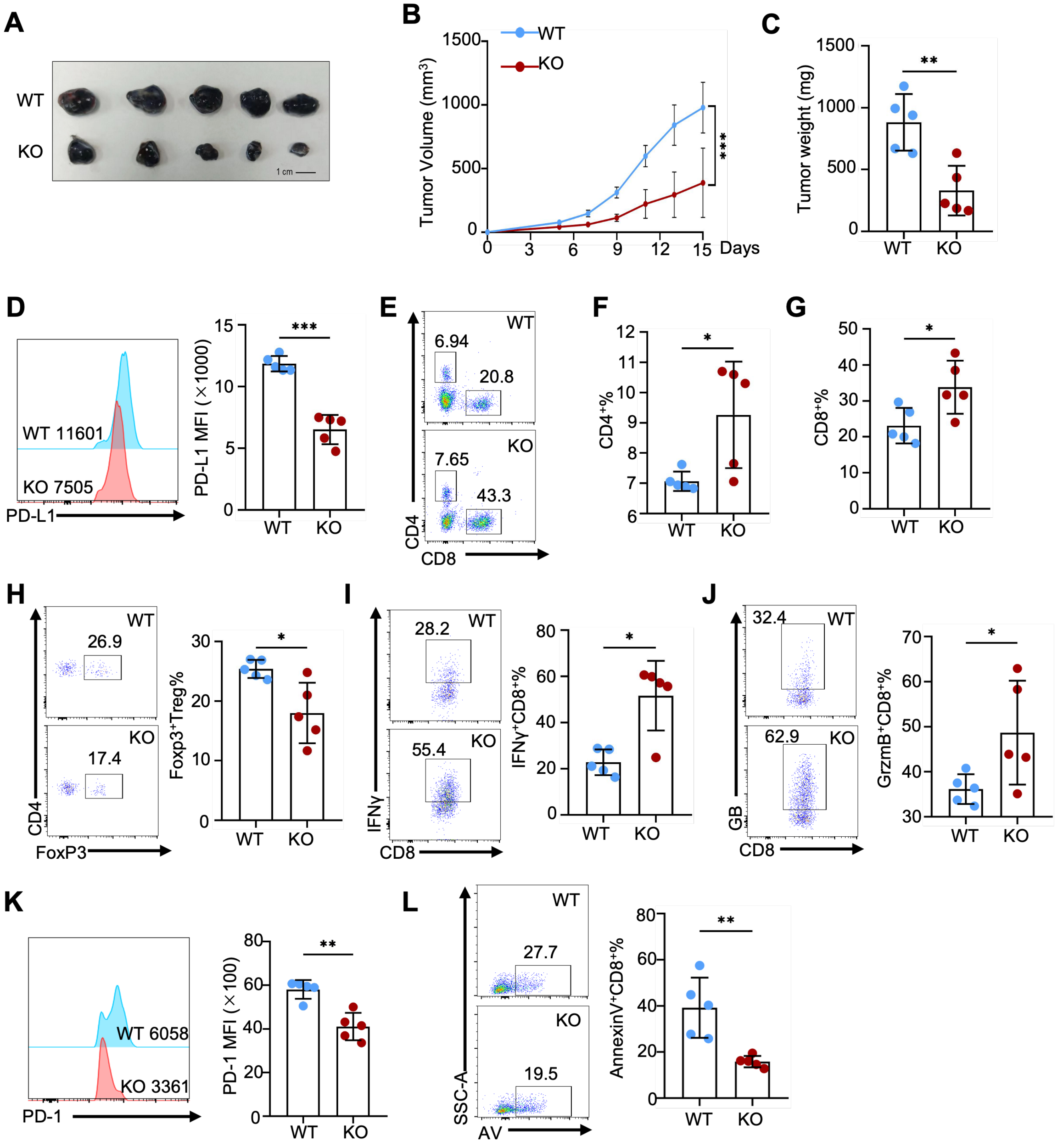

Fig. S11. SIRT2 knockout enhances antitumor immunity in B16 melanoma. (**A-C**) WT and SIRT2 KO B16 cells were subcutaneously implanted into the right flank of C57BL/6J mice (n=5). Representative images of mouse tumors (A), tumor volume (B) and tumor weight (C). (**D**) Representative flow cytometry plots of B16 tumors. (**E**) Quantification of surface PD-L1 on B16 tumors (n = 5). (**F&G**) Quantification of $CD4^+$ (F) and $CD8^+$ (G) T cells represented as percentage of tumor-infiltrating lymphocytes in B16 tumors (n=5). (**H**) Quantification of Treg cells represented as percentage of tumor-infiltrating $CD4^+$ T cells in B16 tumors (n=5). (**I-L**) Quantification of $IFN\gamma^+$ (I) and Granzyme B^+ (Grzm B) (J), PD-1^+ (K) and Annexin V^+ (L) represented as percentage of tumor-infiltrating $CD8^+$ T cells in B16 tumors (n=5). Statistical significance was performed by two-tailed unpaired t test or one-way ANOVA test. *$P < 0.05$; **$P < 0.01$; ***$P < 0.001$; ns, not significant.

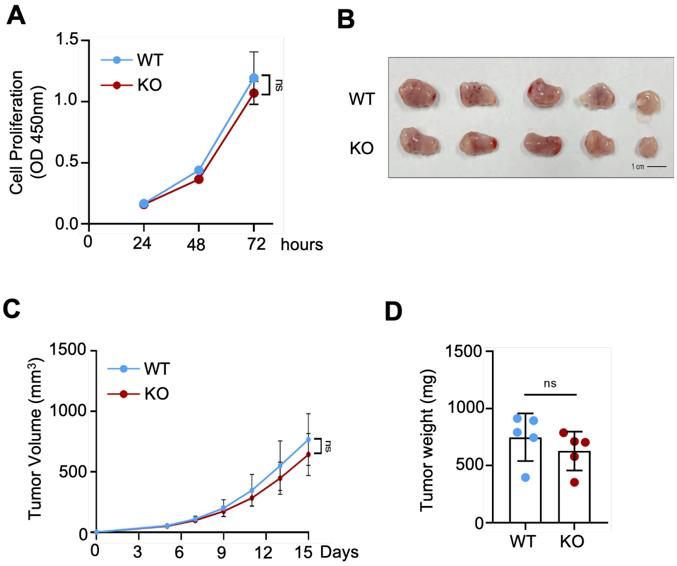

Fig. S12. The effects of SIRT2 knockout on cancer cell growth. (**A**) CCK-8 assay analysis of the effect of SIRT2 knockout on MB49 proliferation. (**B-D**) WT Cand SIRT2 KO MB49 cells were subcutaneously implanted into the right flank of BALB/c nude mice (n=5). Representative images of mouse tumors (B), tumor volume (C) and tumor weight (D). Statistical significance was performed by two-tailed unpaired t test. **P* < 0.05; ***P* < 0.01; ****P* < 0.001; ns, not significant.

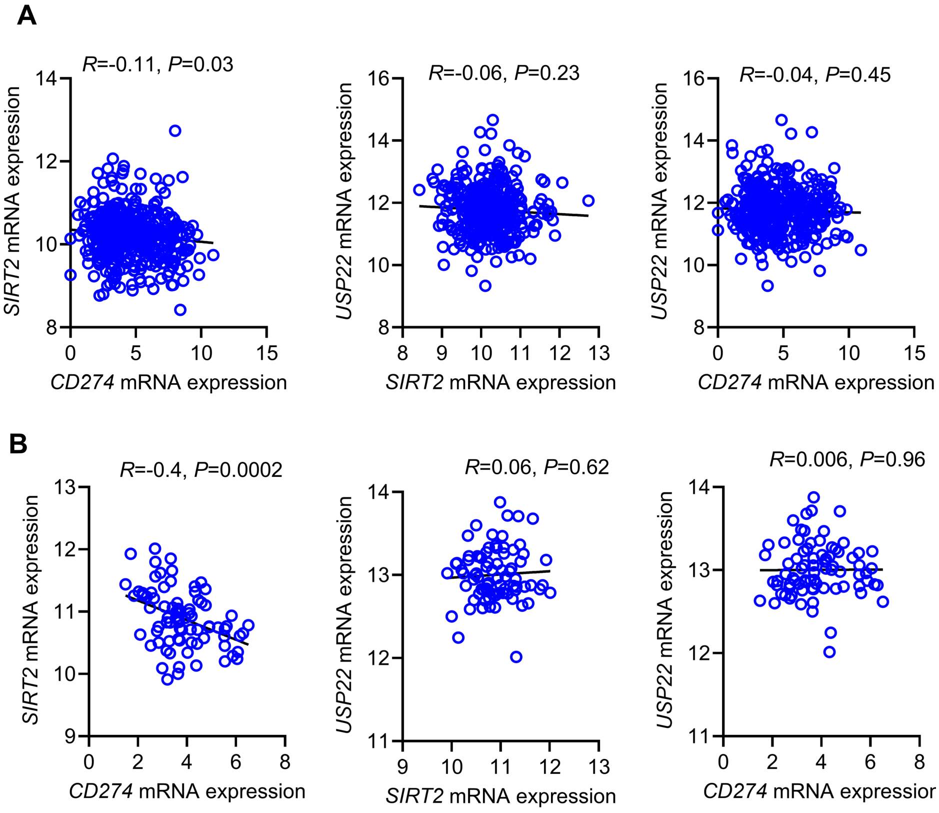

Fig. S13. Analysis of the correlation by Pearson correlation analysis among Sirt1, USP22 and PD-L1 (CD274) correlations in blader cancer (**A**) and melanoma (**B**) using the TCGA data base.

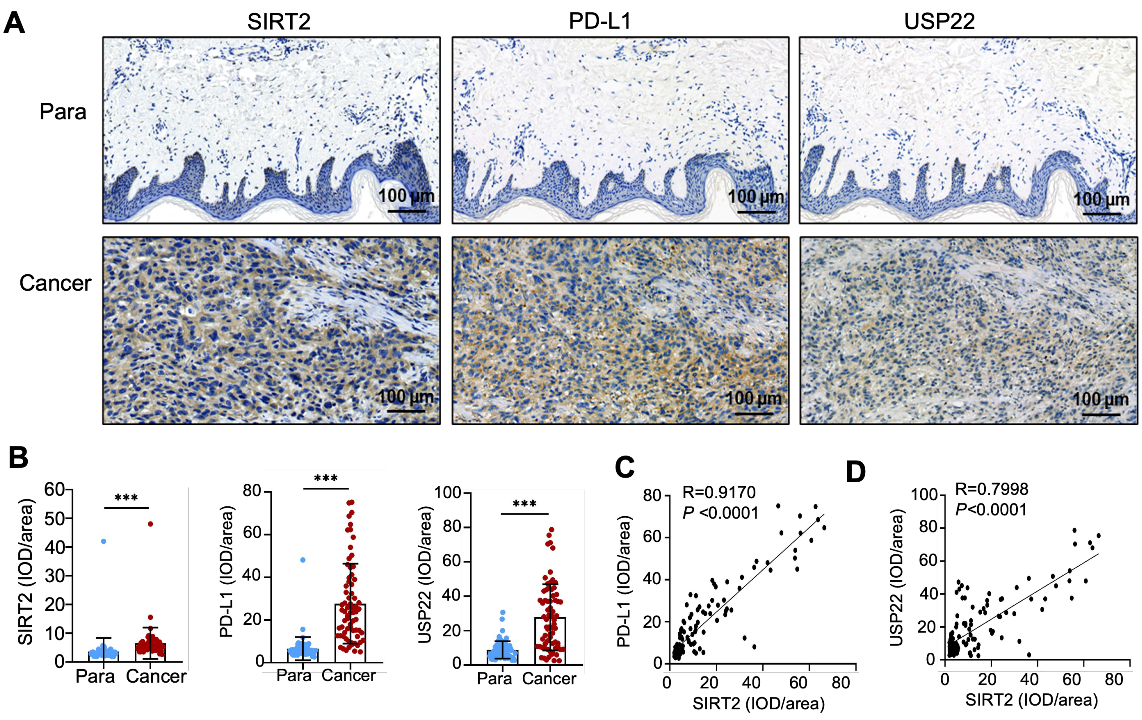

Fig. S14. Positive correlation of SIRT2 with PD-L1 expression in human cancer. (A) Representative IHC staining images of SIRT2, PD-L1, and USP22 in human melanoma and adjacent tissues. Scale bars, 200 µm. **(B)** Quantification of SIRT2, PD-L1, and USP22 staining intensities in melanoma samples (n = 70). **(C, D)** Correlation analysis of SIRT2 with PD-L1 (C) and USP22 (D) in melanoma (n = 70). Statistical significance was determined using a two-tailed unpaired t-test or Pearson correlation analysis. * $P < 0.05$; ** $\boldsymbol{P} < 0.01$; *** $\boldsymbol{P} < 0.001$. ns, not significant.

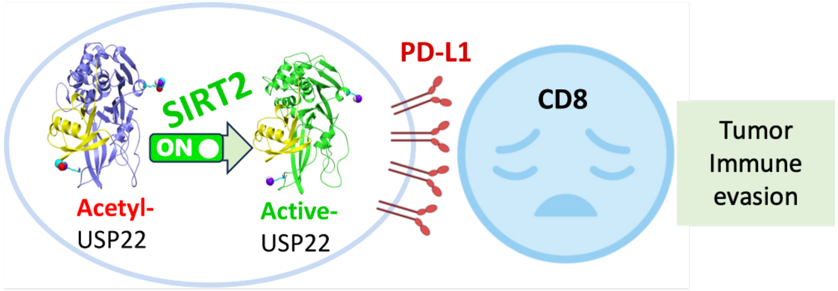

Fig. s15. Proposed working model of SIRT2-mediated PD-L1 upregulation in cancer.

Table s1. Binding Free Energies of SIRT2 and USP22 Protein Model

ΔE_{vdW}	ΔE_{ele}	ΔG_{PB}	ΔG_{SA}	$\Delta G_{binding}$
-850.862	-5063.954	1374.207	-105.233	-4642.321
All the energies are in kJ/mol. Snapshots extracted from the last 100ps MD simulation were submitted to MMPBSA.py for the free energy calculation.				

Table s2. Binding Free Energies of UB and USP22 Lysine Acetylation Protein Model

Protein Model	ΔE_{vdW}	ΔE_{ele}	ΔG_{PB}	ΔG_{SA}	$\Delta G_{binding}$
K505 & K382	-819.35	-2367.05	1826.84	--84.03	-1441
K505-Ac	--805.42	-2041.15	1735.00	-83.95	-1194.93
K382-Ac	-595.06	-1997.88	1428.26	-63.62	-1205.11
K505-Ac & K382-Ac	-740.68	-1868.72	1711.81	-80.5	-974.55
Snapshots extracted from the last 0.5 ns MD simulation were submitted to MMPBSA.py for the free energy calculation. All the energies are in kJ/mol.					

Table S3. Antibody used in the study.

Antibodies reagent	Source	Catalog
USP5	Proteintech	10473-1-AP
USP7	Proteintech	66514-1-Ig
USP8	Proteintech	27791-1-AP
USP9X	Proteintech	55054-1-AP
USP20	Proteintech	17491-1-AP
USP19	Proteintech	25768-1-AP
USP2	Proteintech	10392-1-AP
USP10	Proteintech	19374-1-AP
CSN5	Proteintech	27511-1-AP
USP22	Abcam	ab195289
SIRT2	Proteintech	66410-1-Ig
PD-L1	Abcam	ab213480
PD-L1	Cell Signaling Technology	13684S
Acetylated Lysine	ThermoFisher	MA1-2021
Ubiquitin	Proteintech	10201-2-AP
HA-tag	Cell Signaling Technology	3724S
His-tag	Cell Signaling Technology	12698S
Flag-tag	Sigma	F-1804
Myc-tag	Cell Signaling Technology	2276S
β-actin	Proteintech	66009-1-lg
GAPDH	Proteintech	10494-1-AP
HRP-conjugated Affinipure Goat Anti-Rabbit IgG(H+L)	Proteintech	SA00001-2
HRP-conjugated Affinipure Goat Anti-Mouse IgG(H+L)	Proteintech	SA00001-1

Table S4. gRNAs or siRNAs used in mouse and human cancer cells.

name	Species	sg-RNA Sequence (5'-3')
SIRT1 sgRNA01	human	AGAGATGGCTGGAATTGTCC
SIRT1 sgRNA02	human	CTCCCCGGCGGGGACGACG
SIRT1 sgRNA03	human	GAAGTGCCTCAGATATTAA
SIRT2	human	CGGGTTTATTACAGGGACAGCAG
SIRT2	mouse	AGAGGCCAGTGGACGGGGAG
SIRT3 sgRNA01	human	GTACGATCTCCCGTACCCCG
SIRT3 sgRNA02	human	CTCTACACGCAGAACATCGA
SIRT3 sgRNA03	human	GGGTCTTTGGCAGACTGTGCG
stable overexpression of PD-L1	mouse	CCTCACATTGCCAAAAGACG
stable overexpression of USP22	mouse	GCAGCTGCTGAGGCACGCAG
si-SIRT1	human	GGAAAUAUAUCCUGGACAATT
si-SIRT2	human	GCCAACCAUCUGUCACUACUUTT
si-SIRT3	human	GCUGGAUUGUGACAUAGGATT
si-SIRT4	human	CAUCCAAGCAUGGUGAUUUUTT
si-SIRT5	human	GGAGAUCCAUGGUAGCUUATT

si-SIRT6	human	AGCGGAAGGUGUGGGAACUTT
si-SIRT7	human	CCUGCCGUGUGAGGCGGAA

Table S5. Primers for real-time RT-PCR analysis.

Primer name (RT-qPCR)	Sequence (5'-3')
h-SIRT1-F	TGGAACAGGTTGCGGGAAT
h-SIRT1-R	CTGGGCACCTAGGACATCGA
h-SIRT2-F	CATCCACCGGCCTCTATGAC
h-SIRT2-R	GACAGATGGTTGGCTTGAACTG
h-SIRT3-F	TGCTTCTGCGGCTCTACACG
h-SIRT3-R	ACGTCAGCCCGAATGTCCTC
h-SIRT4-F	CAACCTGCGTTCAATGTGGAG
h-SIRT4-R	GCCAAGTCATCCGACCGTGT
h-SIRT5-F	TGGTCATCACCCAGAACATCG
h-SIRT5-R	TTCTCAGCCACAACTCCACAAG
h-SIRT6-F	TGTGGAAGAATGTGCCAAGTGT
h-SIRT6-R	AGCGATGTACCCAGCGTGAT
h-SIRT7-F	CAGAAAGGGAGAAGCGTTAGTGC
h-SIRT7-R	ACCCGCACGTACTCCCTGTT
h-ACTB-F	GGGAAATCGTGCGTGACATT
h-ACTB-R	GGAACCGCTCATTGCCAAT
h-USP22-F	CGCAAGGCCAAGTCCTGTAT
h-USP22-R	CAGATCAATGGCCAGGTTGTG
h-PD-L1-F	AATTTTGGTTGTGGATCCAGTCA
h-PD-L1-R	AGCTTCTCCTCTCTCTTGGAATTG
m-SIRT2-F	AACATCCGGAACCCTTCTTTG
m-SIRT2-R	CCACTCGTTCCAGCGTGTCT
m-USP22-F	GCTCCCCACACATTCCATACA
m-USP22-R	TCTTCCCATTGTCATCACCTTTG
m-PD-L1-F	GCGAATCACGCTGAAAGTCA
m-PD-L1-R	ACGGGTTGGTGGTCACTGTT
m-ACTB-F	ACTGCCGCATCCTCTTCCT
m-ACTB-R	TCAACGTCACACTTCATGATGGA
