# Supplementary material for: SIRT2-mediated deacetylation activates USP22 catalytic function for PD-L1 protein stabilization and tumor immune escape
Source: J Clin Invest. 2026 Jun 2;136(14):e198270. doi: 10.1172/JCI198270 (PMC13367968; doi:10.1172/JCI198270)

Figure. 1

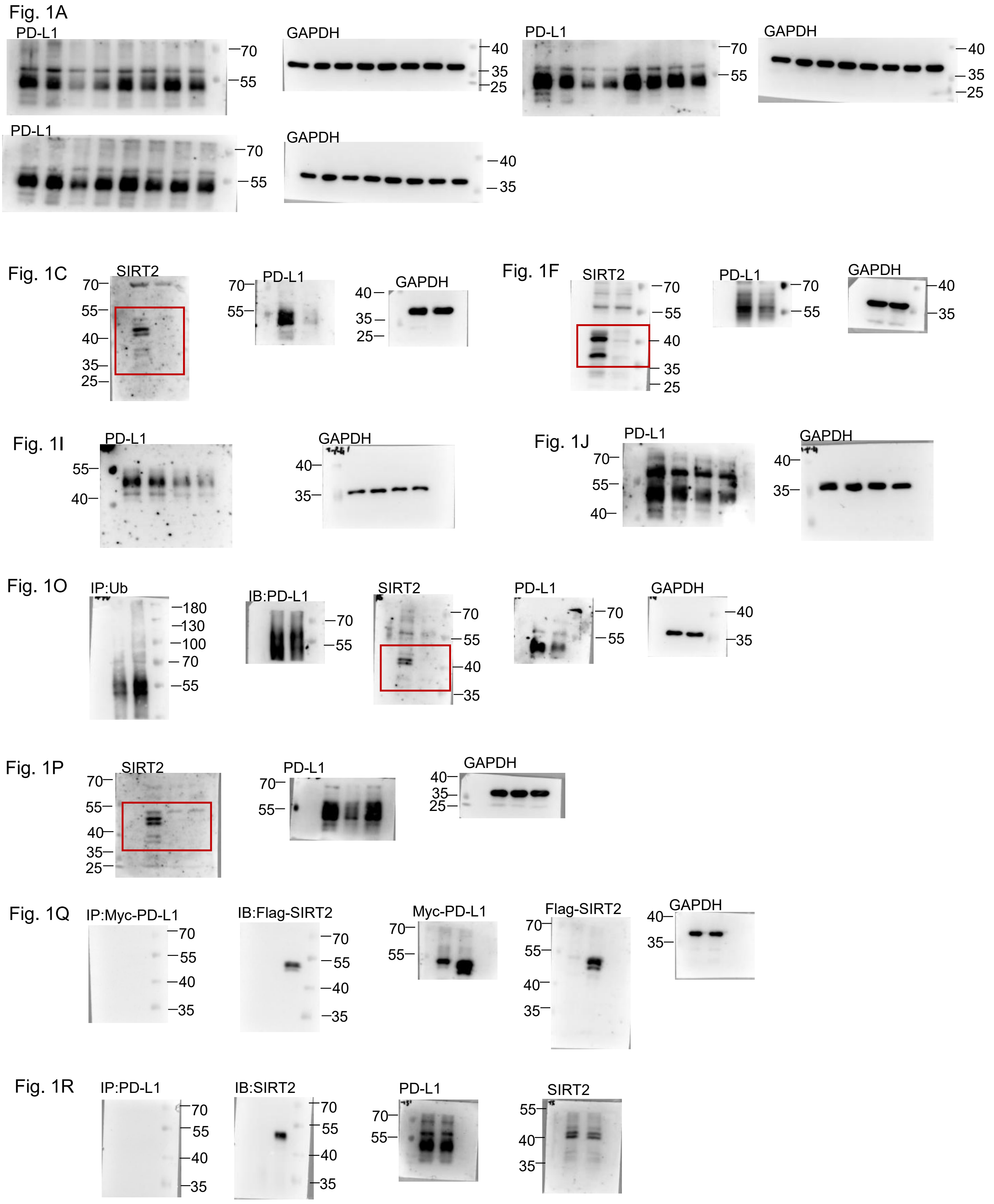

Fig. 1A
PD-L1
GAPDH
PD-L1
GAPDH
PD-L1
GAPDH
Fig. 1C
SIRT2
PD-L1
GAPDH
Fig. 1F
SIRT2
PD-L1
GAPDH
Fig. 1I
PD-L1
GAPDH
Fig. 1J
PD-L1
GAPDH
Fig. 1O
IP:Ub
IB:PD-L1
SIRT2
PD-L1
GAPDH
Fig. 1P
SIRT2
PD-L1
GAPDH
Fig. 1Q
IP:Myc-PD-L1
IB:Flag-SIRT2
Myc-PD-L1
Flag-SIRT2
GAPDH
Fig. 1R
IP:PD-L1
IB:SIRT2
PD-L1
SIRT2

Figure. 2

Fig. 2A

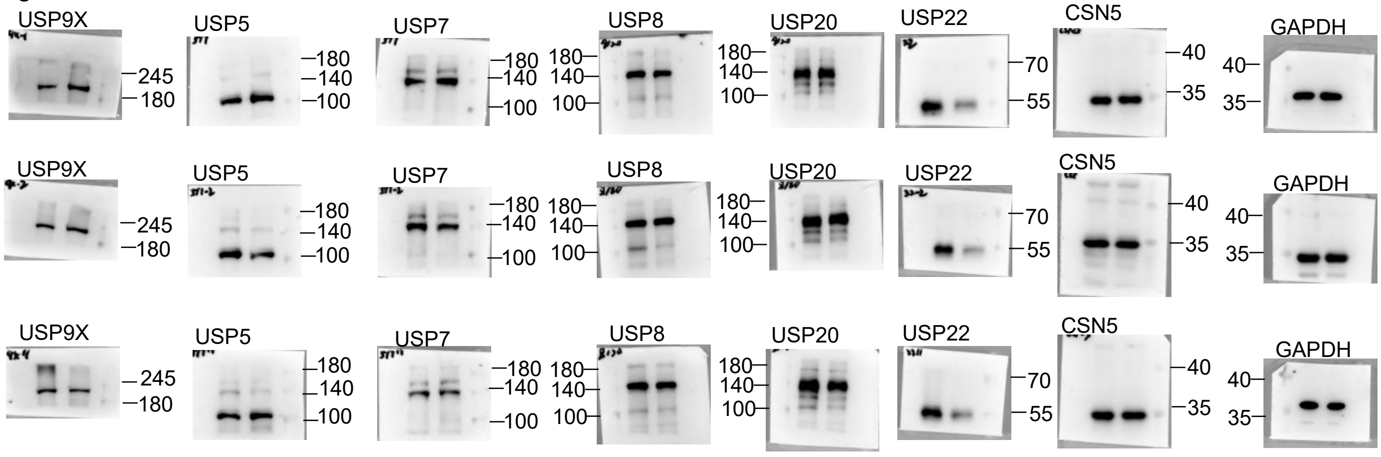

Fig. 2C

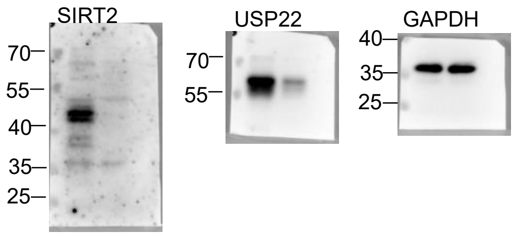

Fig. 2D

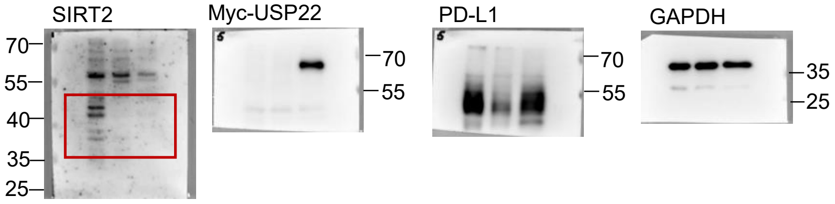

Fig. 2H

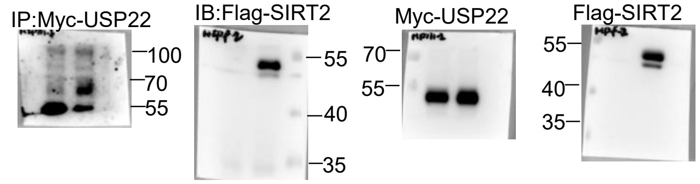

Fig. 2I

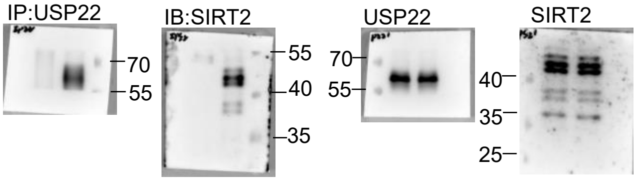

Fig. 2L

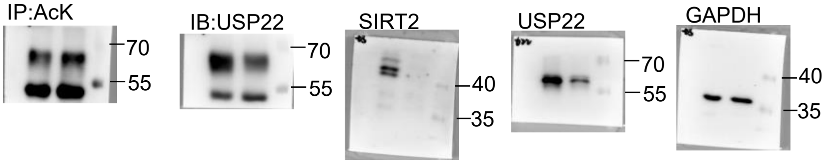

Fig. 2M

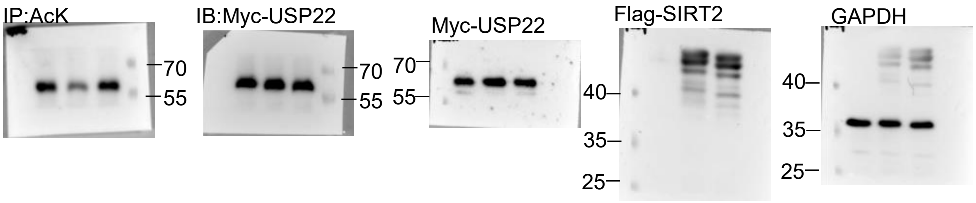

Fig. 2N

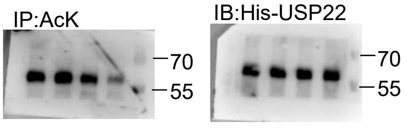

Figure. 3

Fig. 3A

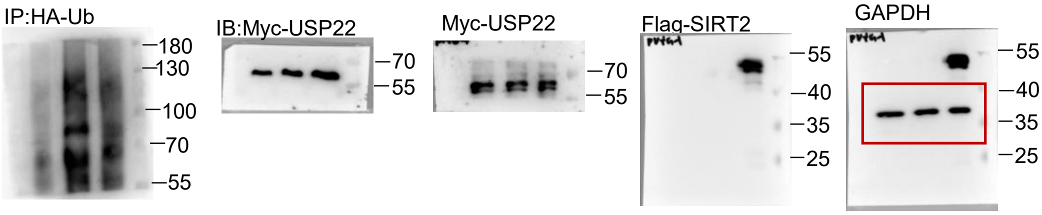

Fig. 3B

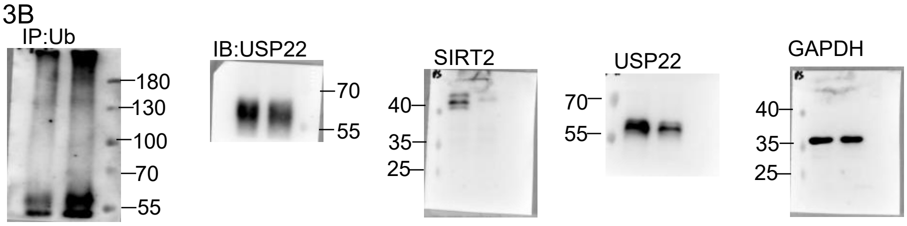

Fig. 3C

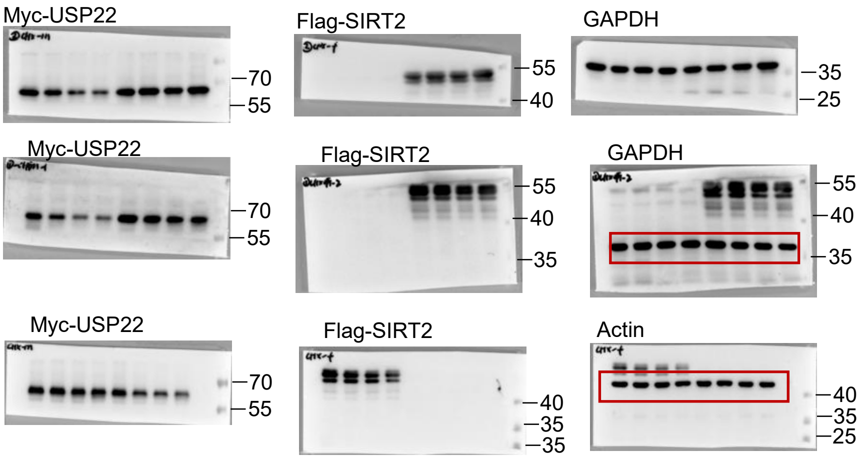

Fig. 3E

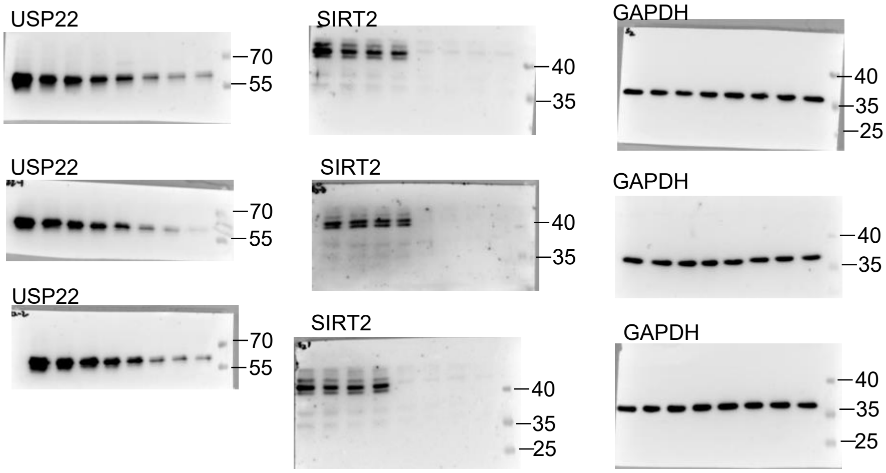

Fig. 3G

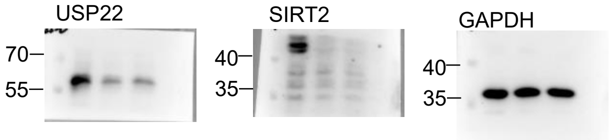

Fig. 3H

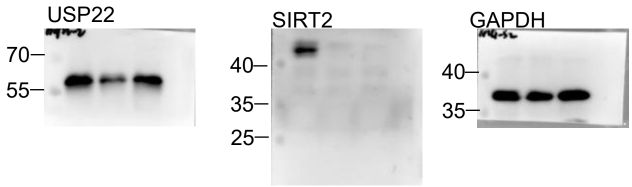

Fig. 3I

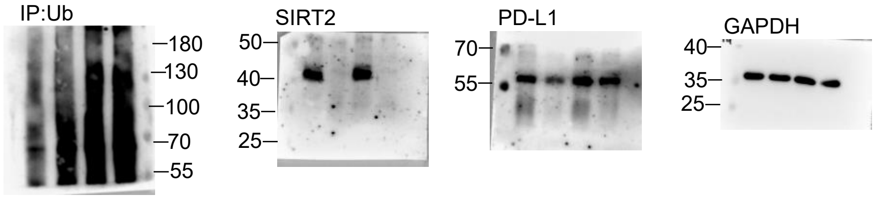

Figure. 4

Fig. 4A

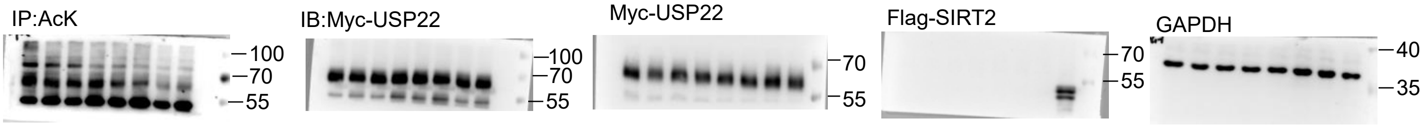

Fig. 4B

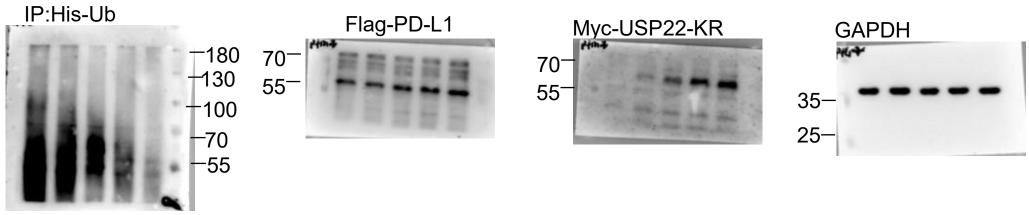

Fig. 4C

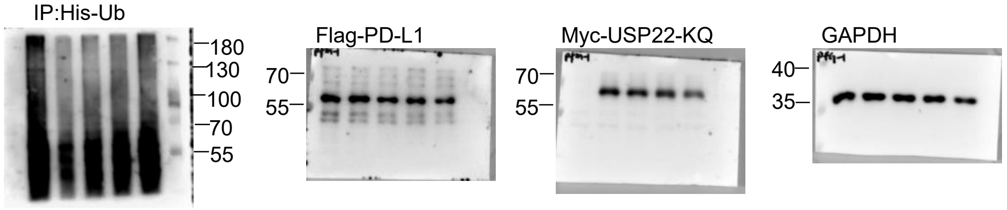

Figure. 5

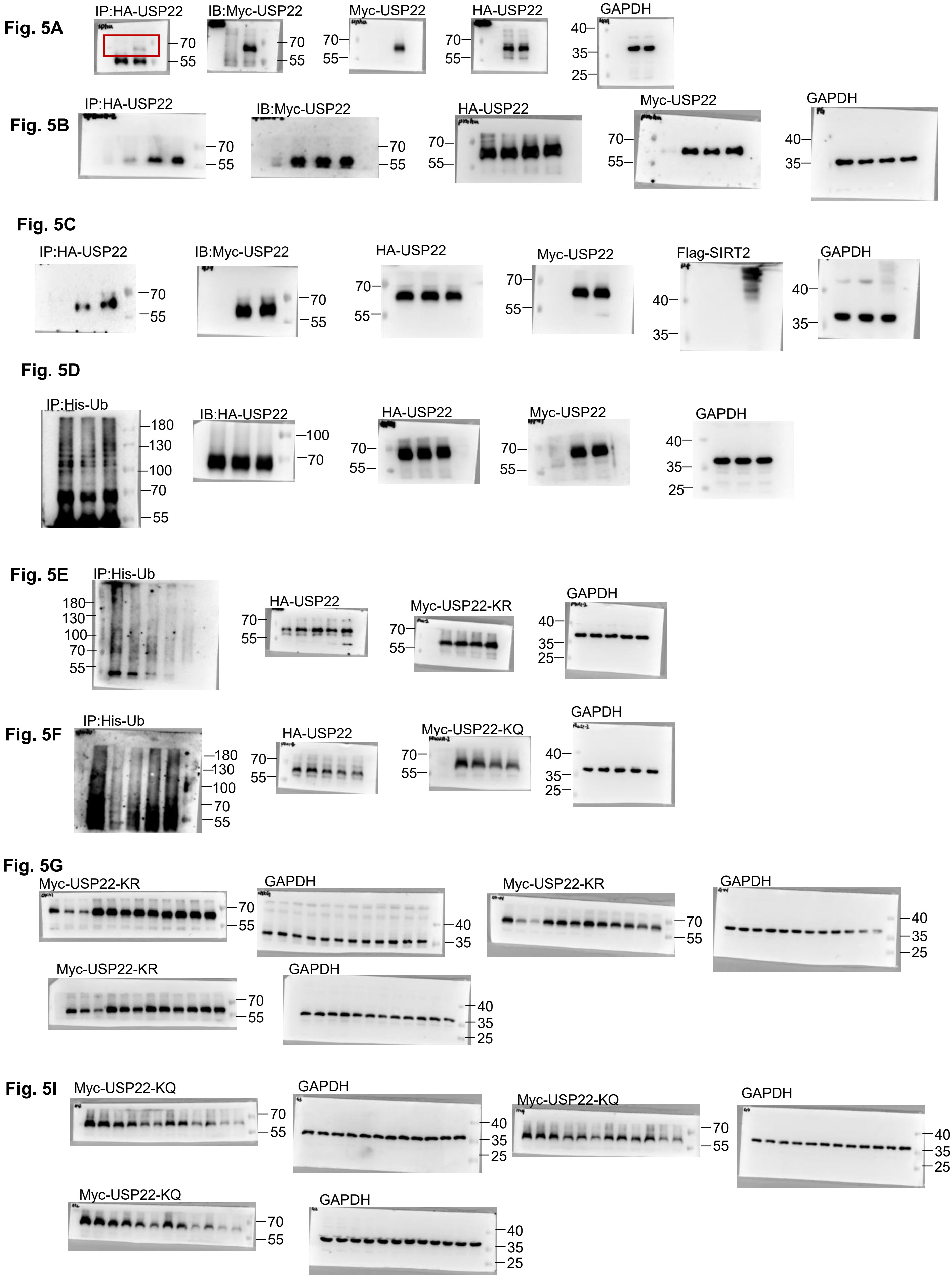
Fig. 5A
IP:HA-USP22
IB:Myc-USP22
Myc-USP22
HA-USP22
GAPDH
70
55
40
35
25
Fig. 5B
IP:HA-USP22
IB:Myc-USP22
HA-USP22
Myc-USP22
GAPDH
Fig. 5C
IP:HA-USP22
IB:Myc-USP22
HA-USP22
Myc-USP22
Flag-SIRT2
GAPDH
Fig. 5D
IP:His-Ub
IB:HA-USP22
HA-USP22
Myc-USP22
GAPDH
180
130
100
Fig. 5E
IP:His-Ub
HA-USP22
Myc-USP22-KR
GAPDH
Fig. 5F
IP:His-Ub
HA-USP22
Myc-USP22-KQ
GAPDH
Fig. 5G
Myc-USP22-KR
GAPDH
Fig. 5I
Myc-USP22-KQ
GAPDH

Fig. 5K

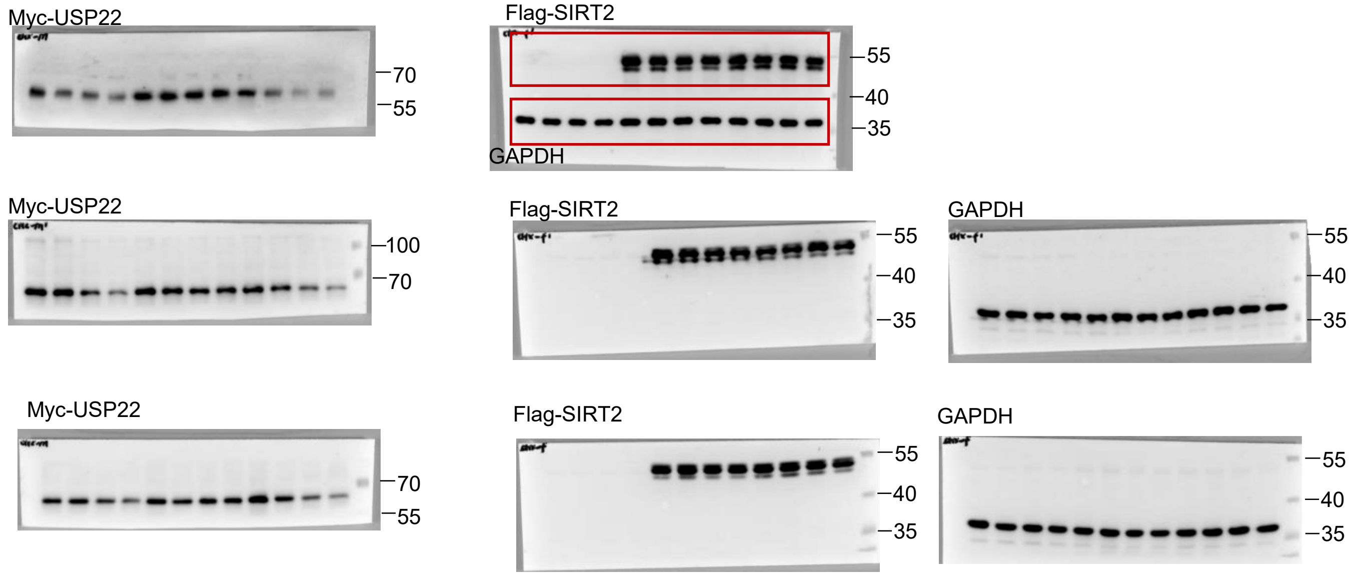

Figure. 7

Fig. 7E

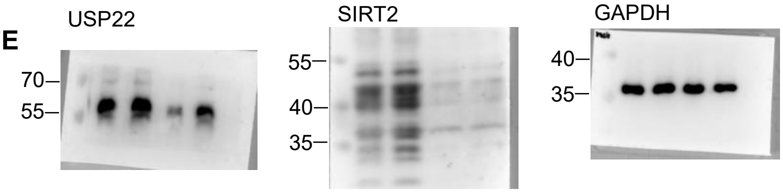

Supplemental Figure

Figure S2A

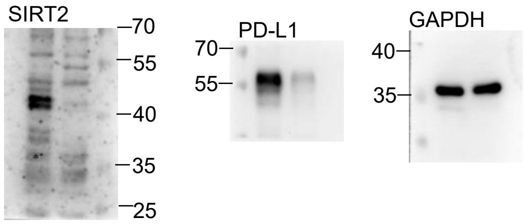

Figure S2F

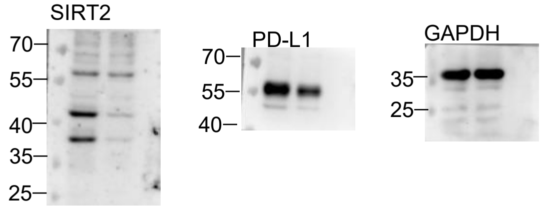

Figure S2K

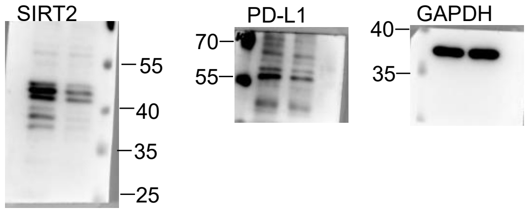

Figure S3

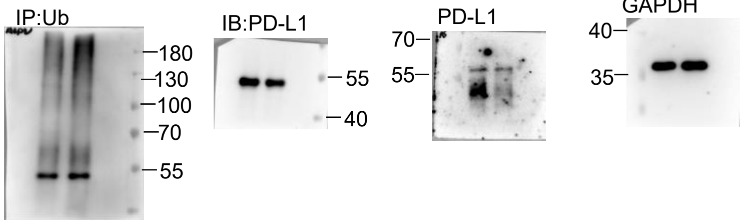

Figure S4A

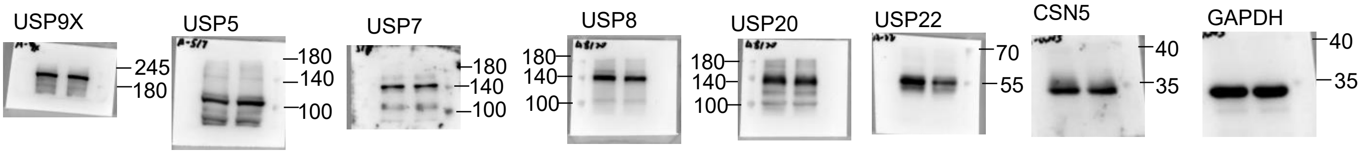

Figure S4B

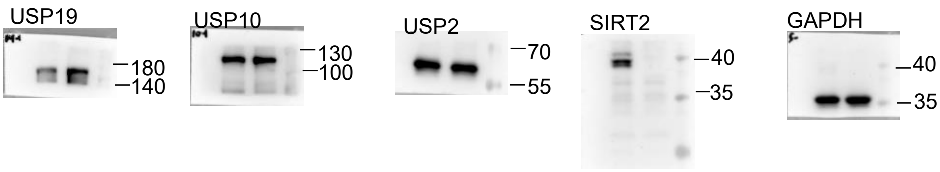

Figure S4C

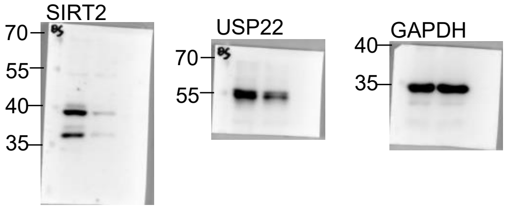

Figure S5A

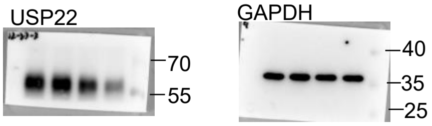

Figure S5B

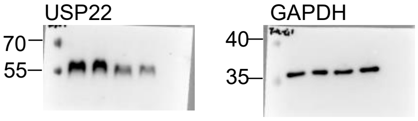

Figure S5C

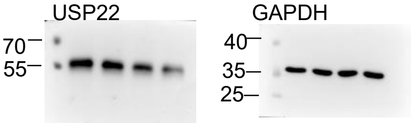

Figure S6A

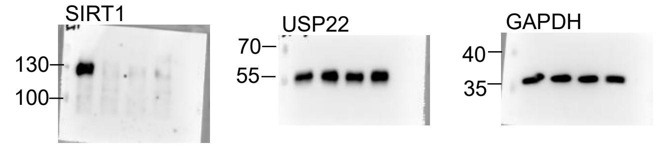

Figure S6B

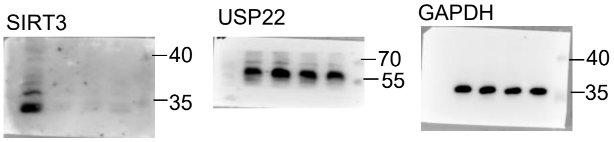

Figure S8A

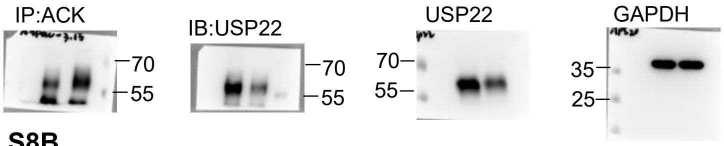

Figure S8B

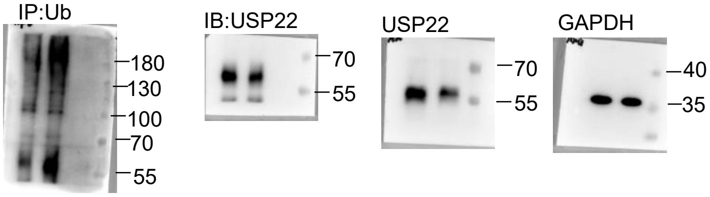

Figure S8C

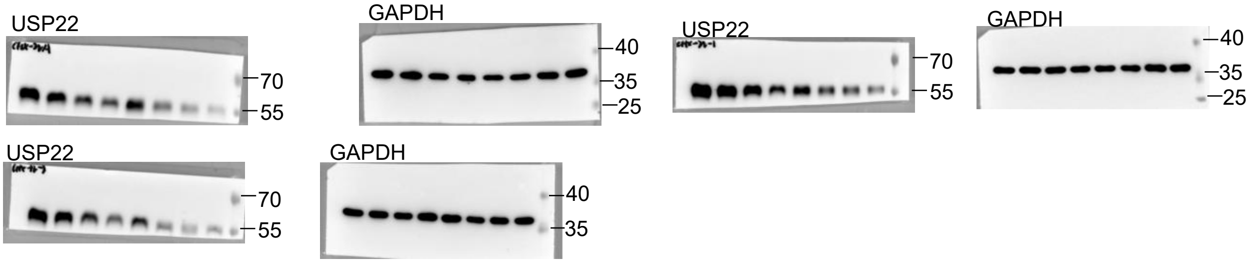

Supplement: Unedited blot and gel images [file jci-136-198270-s183.pdf]
